# DNA Repair Cofactors ATMIN and NBS1 Are Required to Suppress T Cell Activation

**DOI:** 10.1371/journal.pgen.1005645

**Published:** 2015-11-06

**Authors:** Jana Prochazkova, Shinya Sakaguchi, Michel Owusu, Abdelghani Mazouzi, Marc Wiedner, Georgia Velimezi, Martin Moder, Gleb Turchinovich, Anastasiya Hladik, Elisabeth Gurnhofer, Adrian Hayday, Axel Behrens, Sylvia Knapp, Lukas Kenner, Wilfried Ellmeier, Joanna I. Loizou

**Affiliations:** 1 CeMM Research Center for Molecular Medicine of the Austrian Academy of Sciences, Vienna, Austria; 2 Division of Immunobiology, Institute of Immunology, Center for Pathophysiology, Infectiology and Immunology, Vienna, Austria; 3 London Research Institute, Cancer Research UK, London, United Kingdom; 4 Department of Medicine I, Laboratory of Infection Biology, Medical University of Vienna, Vienna, Austria; 5 Clinical Institute for Pathology, Medical University Vienna, Vienna, Austria; Institute for Research in Biomedicine (IRB Barcelona), SPAIN

## Abstract

Proper development of the immune system is an intricate process dependent on many factors, including an intact DNA damage response. The DNA double-strand break signaling kinase ATM and its cofactor NBS1 are required during T cell development and for the maintenance of genomic stability. The role of a second ATM cofactor, ATMIN (also known as ASCIZ) in T cells is much less clear, and whether ATMIN and NBS1 function in synergy in T cells is unknown. Here, we investigate the roles of ATMIN and NBS1, either alone or in combination, using murine models. We show loss of NBS1 led to a developmental block at the double-positive stage of T cell development, as well as reduced TCRα recombination, that was unexpectedly neither exacerbated nor alleviated by concomitant loss of ATMIN. In contrast, loss of both ATMIN and NBS1 enhanced DNA damage that drove spontaneous peripheral T cell hyperactivation, proliferation as well as excessive production of proinflammatory cytokines and chemokines, leading to a highly inflammatory environment. Intriguingly, the disease causing T cells were largely proficient for both ATMIN and NBS1. *In vivo* this resulted in severe intestinal inflammation, colitis and premature death. Our findings reveal a novel model for an intestinal bowel disease phenotype that occurs upon combined loss of the DNA repair cofactors ATMIN and NBS1.

## Author Summary

Defects in DNA repair pathways can lead to pathogenesis within the immune system, an example of which is inflammatory bowel disease (IBD). Yet the underlying genetic causes of IBD are often unclear. The DNA repair kinase ATM is crucial for the proper development and function of the immune system. ATM is regulated in a stimulus dependent manner by its cofactors, ATMIN and NBS1. These cofactors compete for ATM binding and in doing so regulate ATM kinase activity. Whereas both ATM and NBS1 function in T cell development and in the maintenance of genomic stability within such cells, the role of ATMIN (and the contribution of ATMIN and NBS1) in T cell function is unknown. Here, we show that whereas NBS1 has distinct ATMIN-independent functions during VDJ recombination, loss of both cofactors resulted in exacerbated DNA damage, T cell hyperactivation, inflammation and an IBD phenotype. The pathology was driven by T cells largely proficient for both ATMIN and NBS1. These data demonstrate additive effects revealed upon loss of both ATMIN and NBS1, thus illustrating the importance of these two DNA repair cofactors in proper T cell development and function.

## Introduction

Defects in T cell development can result due to inefficient repair of DNA lesions that are generated in a programmed manner during the recombination of variable, diverse and joining (VDJ) gene segments, a process that is crucial for the generation of the T cell receptor (TCR) [1]. Therefore, proper repair of such breaks is vital for lymphocyte generation and survival. An important kinase that functions in the repair of such DNA lesions is Ataxia Telangiectasia Mutated (ATM) [2]. Patients (known as AT patients) and mice deficient for ATM show T and B cell developmental defects and lymphoma generation [3–11]. Although the development of thymic lymphoma has been linked to aberrant TCR recombination [11,12], it has also been proposed that oxidative damage plays an important part in generating these tumors [13,14]. In line with this hypothesis, treatment of ATM-deficient mice with scavengers of reactive oxygen species (ROS) alleviates the lymphocyte developmental defects observed in these mice, as well as the development of thymic lymphomas [13].

ATM is regulated by its cofactor NBS1, mutated in Nijmegen Breakage Syndrome, following the generation of DNA double-strand breaks [15,16]. NBS1 functions as part of the MRN complex, consisting of MRE11, RAD50 and NBS1, that is a major sensor of DNA double-strand breaks [2,17]. The MRN complex binds to broken DNA ends and induces ATM activation to repair the DNA lesions [17]. Recently, however, the MRN complex has also been linked to activating another kinase that belongs to the ATM superfamily known as ATR (for ataxia telangiectasia and Rad3 related) [18–22]. The role of ATR is to resolve replication stress by binding single-stranded DNA [23]. Thus, MRN participates in the activation of ATM and ATR. Within the immune system, loss of NBS1 leads to defects in T and B cell development characterized by lymphopenia [24–27]. Nijmegen Breakage Syndrome patients are also predisposed to malignancies, particularly those of the lymphoid system [28]. Furthermore, a ‘humanized’ NBS1 mouse model has been generated and as well as displaying immunodeficiency, this model also develops T cell lymphoma, in a p53 dependent manner [27].

ATM has also been shown to be regulated by a second cofactor, ATMIN (for ATM Interactor) [29] also known as ASCIZ (ATM substrate Chk2-interacting Zn^2+^-finger protein) [30]. It is known that ATMIN functions in resolving DNA damage. ATMIN has been reported to function as an ATM-cofactor following replicative stress and hypotonic stress [29,31]. It is also required to localize RAD51 following DNA methylation damage [30]. Furthermore, in the ageing mouse brain ATMIN-deficient mice accumulate oxidative DNA damage [32] and in B cells loss of ATMIN during later stages of development (the pro B cell stage) leads to genomic instability, chromosomal translocations and tumourigenesis [33]. Yet the functions of ATMIN are not limited to DNA repair: in B cells ATMIN also functions as a transcription factor where it is required to regulate the expression of DYNLL1 [34]. Loss of ATMIN during early B cell development leads to increased apoptosis due to reduced DYNLL1 expression hence inducing Bim-dependent apoptosis [34].

Mechanistically, it has been shown that NBS1 and ATMIN compete for ATM binding and hence regulate ATM function [35]. ATM activity following DNA double-strand breaks is increased in ATMIN mutant cells whereas ATMIN-dependent ATM signaling is increased in cells deficient for NBS1 [35]. Hence, the absence of one cofactor increases activity through the alternative pathway. Because of this mechanism of ATM regulation, ATMIN deficiency rescues NBS1-dependent cellular lethality [35].

Mutations in DNA repair genes, including ATM and NBS1 have been linked with immunodeficiencies in patients and furthermore immune deficiency is an important factor in causality of human inflammatory diseases such as inflammatory bowel disease (IBD) [28,36,37]. For example, patients with Omenn syndrome or common variable immunodeficiency (CVID) carry hypomorphic mutations in RAG1/2, the enzymes that initiate recombination in B and T cells [38–40]. These patients suffer from immunodeficiency but also from chronic inflammation affecting multiple tissues including the gut [38,41]. This is, in some cases, due to abnormal T cell production that displays increased affinity for self-antigens. T cells from these patients are autoreactive and hence give rise to chronic inflammation. In addition, it was recently shown that unrepaired lesions in AT patients induce a type I interferon response, which leads to inflammatory manifestations [42]. However, the underlying genetic causes for such inflammatory diseases, including IBD, are largely not known.

In order to address whether loss of ATMIN, alone or in combination with loss of NBS1, leads to T cell-related defects and pathologies, we generated murine models with deletion of ATMIN and NBS1 either alone or in combination. We show that loss of NBS1 led to a developmental block at the double-positive (DP) stage of T cell development and reduced TCRα recombination. Unexpectedly, these developmental functions of NBS1 were neither exacerbated nor alleviated by concomitant loss of ATMIN. In contrast, compound mutant mice lacking both ATMIN and NBS1 in T cells displayed immune hyperactivation and an IBD phenotype, which is mediated by T cells and transplantable into control mice. ATMIN/NBS1-deficient mice carried higher levels of DNA damage and their T cells produced elevated levels of proinflammatory cytokines, coupled with increased proliferation. This generated a proinflammatory environment in the intestine, as well as the spleen, leading to premature death. However, the pathology-causing T cells were found to be largely proficient for both ATMIN and NBS1.

## Results

### NBS1 is required for T cell development and TCRα recombination, independently of ATMIN

**Fig 1:**
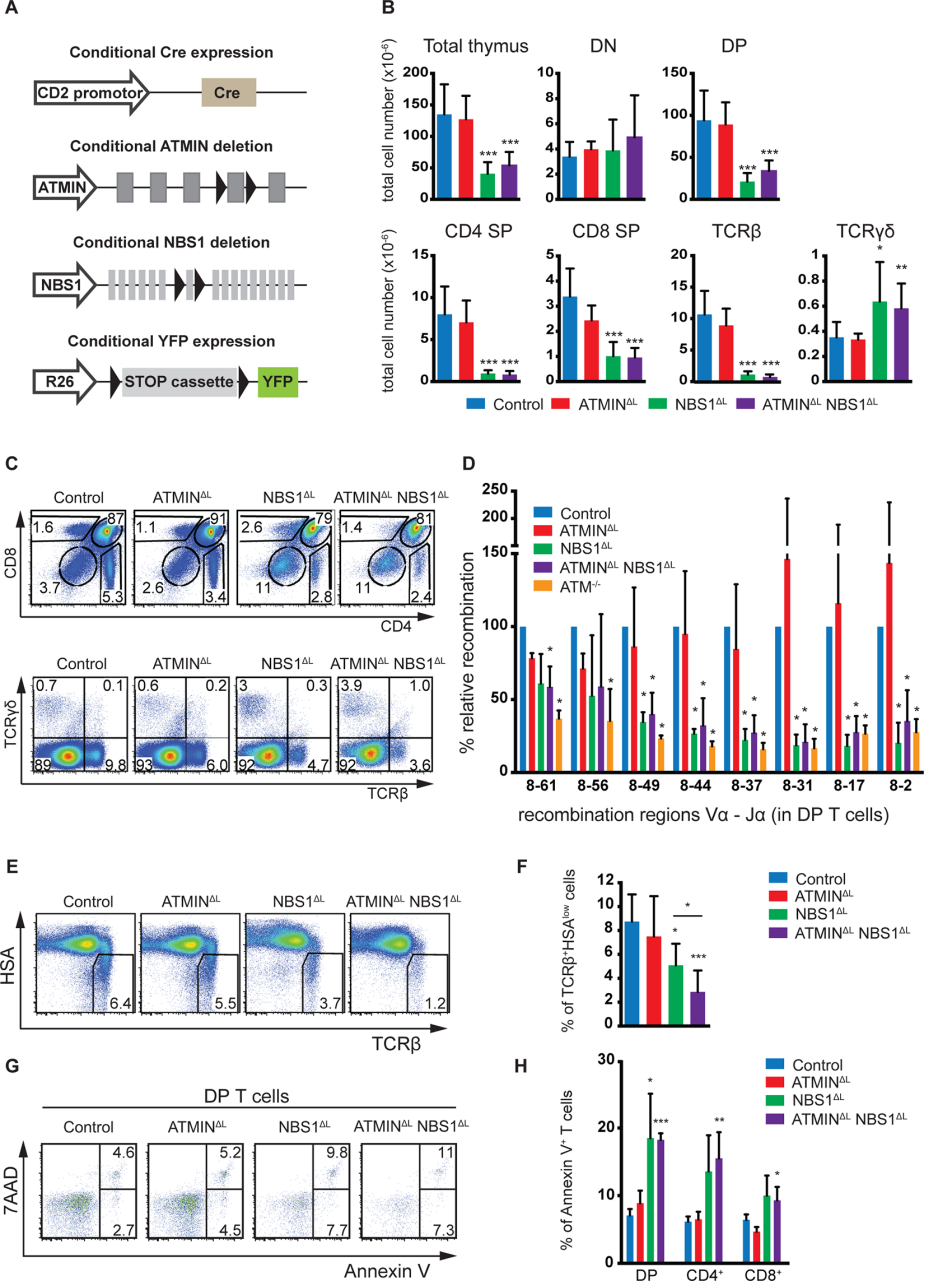
NBS1 is required for T cell development and TCRα recombination, which is largely unaffected by concomitant loss of ATMIN. (A) Schematic representation of the strategy to conditionally delete ATMIN and/or NBS1. Exon 4 of ATMIN, exon 6 of NBS1 and a ‘stop’ cassette inserted upstream of the YFP reporter gene (on the Rosa26 locus, denoted as ‘R26’) were flanked by LoxP sites (denoted by arrow heads). Cre recombinase was expressed under the control of the CD2 promotor. (B) Total thymic cellularity and thymic numbers of double-negative (DN; CD4^-^CD8^-^), double-positive (DP; CD4^+^CD8^+^), CD4 single-positive (SP; CD4^+^CD8^-^), CD8 single-positive (SP; CD4^-^CD8^+^), TCRβ^+^ (TCRβ^+^ TCRγδ^-^) and TCRγδ (TCRβ^-^ TCRγδ^+^) cells in control, ATMIN^ΔL^, NBS1^ΔL^ and ATMIN^ΔL^NBS1^ΔL^ mice. N = 4–10 mice per genotype. (C) Representative flow cytometry plots for B. (D) Quantitative PCR analysis of eight Vα8-Jα recombination regions in purified DP thymic T cells from control, ATMIN^ΔL^, NBS1^ΔL^, ATMIN^ΔL^NBS1^ΔL^ and ATM^-/-^ mice. Results are normalized to the control DP thymic T cells. N = 3 mice per genotype. (E) Representative flow cytometry plots of TCRβ^+^HSA^low^ mature thymocytes isolated from control, ATMIN^ΔL^, NBS1^ΔL^ and ATMIN^ΔL^NBS1^ΔL^ mice. N = 5–7 mice per genotype (F) Quantification of E. (G) Representative flow cytometry data of apoptotic and necrotic (Annexin V^+^ and Annexin V^+^ 7AAD^+^, respectively) DP thymocytes in mice indicated in E. N = 4–7 mice per genotype (H) Quantification of Annexin V^+^ cells within DP, CD4^+^ and CD8^+^ SP thymocytes. Error bars represent SEM (*P<0.05, **P<0.01, ***P<0.001).

To determine the contribution of ATMIN and NBS1 in T cell development and function, we generated mice lacking ATMIN (ATMIN^ΔL^), NBS1 (NBS1^ΔL^) or both ATMIN and NBS1 (ATMIN^ΔL^NBS1^ΔL^), in T lymphocytes by crossing ‘floxed’ mice [33,43] to CD2-cre expressing mice [44] (denoted as ΔL for ‘lymphocyte’) (Fig 1A). These mice were then intercrossed with mice that expressed YFP in CD2-cre expressing cells, from the ROSA26 locus [45] (Fig 1A). The efficiency of deletion of ATMIN and/or NBS1 was determined by PCR, performed on DNA from thymus and spleen (S1A Fig). We additionally confirmed the deletion of ATMIN and NBS1 (as well as ATM) at the protein level in the thymus and the spleen (S1B and S1C Fig). Deletion achieved with CD2-cre was minimal in the spleen, since this tissue is not made up exclusively of T cells.

CD2-cre mediated deletion of NBS1 led to a severe reduction in thymic cellularity by >50% and this surprisingly was neither exacerbated nor alleviated by co-deletion of ATMIN (Fig 1B). Moreover, NBS1^ΔL^ mice displayed decreased CD4, CD8 double-positive (DP) T cells and CD4 single-positive (SP) as well as CD8 SP cells (Fig 1B and 1C) and these phenotypes were not alleviated by concomitant loss of ATMIN. The cre recombinase was expressed from the double-negative (DN) stage of T cell development (S2A Fig) and efficiently through DN1 to DN4 stages of T cell development (S2B Fig). This in turn led to efficient deletion of NBS1 in DN1-4 stages of T cell development (S2C Fig). We observed an increase in the total number and percentage of TCRγδ^+^ T cells upon loss of NBS1 (Fig 1B and 1C and S3A Fig). Loss of ATMIN alone during T cell development did not lead to an apparent phenotype.

The NBS1-dependent block in T cell development was also apparent on the periphery, as indicated by a decrease in the total number of splenocytes in mice deficient for NBS1 (S3B Fig). Similarly, the relative percentage of CD4^+^, CD8^+^ and TCRβ^+^ T cells was dramatically reduced in the spleens of NBS1^ΔL^ and ATMIN^ΔL^NBS1^ΔL^ mice (S3B and S3C Fig).

Since both ATM and NBS1 are required for the repair of DNA double-strand breaks, a process that occurs during VDJ recombination, we investigated whether recombination of the TCRβ locus was affected in NBS1^ΔL^ and ATMIN^ΔL^NBS1^ΔL^ mice. Southern blotting of two distinct TCRβ recombination regions did not reveal a difference in efficiency of TCRβ recombination in mice lacking ATM, ATMIN, NBS1 or both ATMIN and NBS1 (S4A Fig). Also, there was no effect on the quantification of DN T cell subpopulations in any of the genotypes (DN1-DN4; S4B and S4C Fig). Annexin V staining did not reveal an increase in the percentage of apoptotic cells in DN1-4 T cells (S4D and S4E Fig) and *in vivo* bromodeoxyuridine (BrdU) labelling did not suggest impairment in the proliferation of DN cells (S4F and S4G Fig). In summary, we did not detect any defects in the DN T cells that would underlie the developmental block at the DP stage in NBS1^ΔL^ mice.

Next, we investigated the efficiency of TCRα recombination by quantitative PCR in DP T cells at 16 different recombination regions in the TCRα locus (represented schematically in S5A Fig). This revealed a requirement for NBS1 in TCRα recombination (for most of the recombination regions we investigated) (Fig 1D and S5B Fig). Indeed, this defect in recombination is similar to that which occurs due to lack of ATM [46]. Hence, these data reveal NBS1, and not ATMIN, to be the ATM cofactor required for TCRα recombination.

Since TCRα recombination is essential for T cell maturation we assessed the proportion of mature HSA^low^TCRβ^+^ T cells in the thymus. We observed a decrease in the percentage of mature cells in the thymi of NBS1^ΔL^ and ATMIN^ΔL^NBS1^ΔL^ mice (Fig 1E and 1F). Annexin V staining revealed elevated apoptotic and necrotic cells in ATMIN^ΔL^NBS1^ΔL^ and NBS1^ΔL^ mice (Fig 1G and 1H). To test whether this could contribute to T cell activation, we measured CD44^+^ cells and found CD44^+^CD4^+^ T cells to be enhanced in the thymi of ATMIN^ΔL^NBS1^ΔL^ (S6A and S6B Fig).

In summary, we reveal novel functions of NBS1 in T cell development since we observe that loss of NBS1 leads to a block in T cell development at the DP stage as well as defective TCRα recombination. Hence, NBS1^ΔL^ mice show reduced mature T cells, and this phenotype is surprisingly exacerbated (and not rescued) upon co-deletion of ATMIN. We also observe increased DNA damage and apoptosis in NBS1^ΔL^ mice, but more so in ATMIN^ΔL^NBS1^ΔL^ mice.

### Concomitant loss of ATMIN and NBS1 leads to increased mortality due to peripheral immune activation

**Fig 2:**
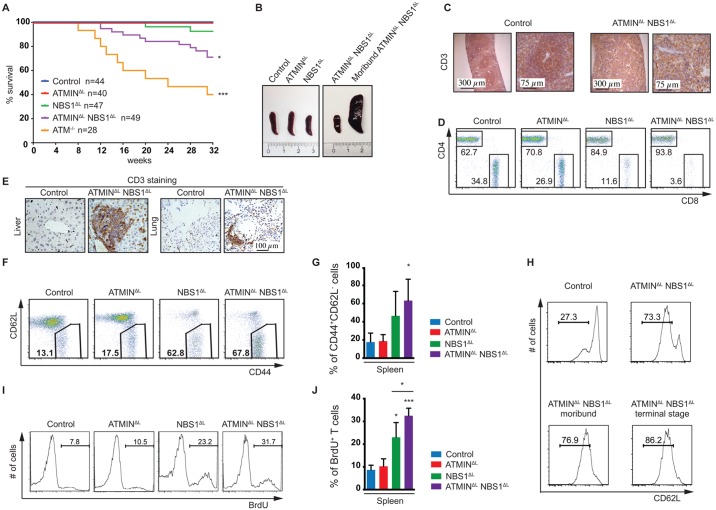
Loss of ATMIN in combination with NBS1, in T cells, leads to increased mortality due to T cell activation. (A) Kaplan-Meier survival curve of control, ATMIN^ΔL^, NBS1^ΔL^, ATMIN^ΔL^NBS1^ΔL^ and ATM^-/-^ mice. Survival was monitored for 32 weeks. (B) Representative images of spleens of control, ATMIN^ΔL^, NBS1^ΔL^ and ATMIN^ΔL^NBS1^ΔL^ mice as well as a moribund ATMIN^ΔL^NBS1^ΔL^ mouse. (C) Histological analysis of the spleen of a control and a moribund ATMIN^ΔL^NBS1^ΔL^ mouse stained for T cells using an anti-CD3 antibody. (D) Representative flow cytometry data of CD4 and CD8 T cells (gated on the TCRβ^+^ population) in the spleen of control, ATMIN^ΔL^, NBS1^ΔL^ and ATMIN^ΔL^NBS1^ΔL^ mice. (E) Histological analysis by using an anti-CD3 antibody to visualize T cells in the liver and lung of control and ATMIN^ΔL^NBS1^ΔL^ mice. (F) Representative flow cytometry data of activated CD62L^low^CD44^+^ T cells (gated on the TCRβ^+^ population) in the spleen of control, ATMIN^ΔL^, NBS1^ΔL^ and ATMIN^ΔL^NBS1^ΔL^ mice. (G) Quantification of F. N = 3–4 mice per genotype. (H) Flow cytometry data showing the percentage of antigen-experienced CD62L^low^CD4^+^ T cells (gated on the TCRβ^+^ population) in the spleen of control and ATMIN^ΔL^NBS1^ΔL^ mice as well as moribund ATMIN^ΔL^NBS1^ΔL^ mice. (I) Representative flow cytometry data of proliferating (BrdU^+^) T cells (gated on the TCRβ^+^ population) in the spleen of mice indicated in F, measured by *in vivo* BrdU incorporation over a period of 4 days. (J) Quantification of I. N = 3–4 mice per genotype. Error bars represent SEM (*P<0.05, ***P<0.001).

ATMIN^ΔL^NBS1^ΔL^ mice but not ATMIN^ΔL^ or NBS1^ΔL^ mice display increased mortality (Fig 2A). Whereas ATM^-/-^ mice developed thymic lymphomas, the compound mutant mice developed splenomegaly marked by an accumulation of CD3^+^ cells (Fig 2B and 2C) that were of a CD4^+^ subtype (Fig 2D). The CD8^+^ T cells were decreased (Fig 2D). An infiltration of CD3^+^ T cells was observed in multiple organs, including the liver and lungs of moribund ATMIN^ΔL^NBS1^ΔL^ mice (Fig 2E).

T cells from the spleens of ATMIN^ΔL^NBS1^ΔL^ (but also to a lesser extent in NBS1^ΔL^) mice showed an activation phenotype where the proportion of CD62L^low^CD44^+^ activated T cells was increased (Fig 2F and 2G). Moreover, this activation phenotype, characterized by the increased proportion of potentially antigen-experienced CD62L^low^CD44^+^ T cells, correlated with the weight loss of ATMIN^ΔL^NBS1^ΔL^ mice (Fig 2H), as such cells were increased in healthy ATMIN^ΔL^NBS1^ΔL^ mice and continued to gradually increase with the progression of splenomegaly and systemic inflammation. We observed increased proliferation of ATMIN^ΔL^NBS1^ΔL^ T cells by *in vivo* BrdU incorporation (Fig 2I) and furthermore we identified the CD62L^low^CD44^+^ T cells to be of a CD4^+^ subset (S7A Fig).

Unexpectedly, however we observed a decrease in the number of splenic T cells that expressed YFP in ATMIN^ΔL^NBS1^ΔL^ mice (S7B Fig). Furthermore, the vast majority of activated CD62L^low^CD44^+^ T cells from ATMIN^ΔL^NBS1^ΔL^ mice did not express YFP (S7C Fig) and the majority of proliferating BrdU^+^ T cells from ATMIN^ΔL^NBS1^ΔL^ mice were not expressing YFP (S7D Fig). To determine whether YFP^-^ T cells were deleted for ATMIN and/or NBS1 we performed a genotyping PCR on FACS sorted T cells (naïve and activated) from a control mouse, YFP^-^ T cells from ATMIN^ΔL^NBS1^ΔL^ mice and YFP^+^ T cells from an ATMIN^ΔL^NBS1^ΔL^ mouse. Approximately 80% of YFP^-^ ATMIN^ΔL^NBS1^ΔL^ T cells were proficient for ATMIN and/or NBS1 (S7E and S7F Fig). As expected YFP^+^ T cells displayed approximately 100% deletion of ATMIN and/or NBS1 (S7E and S7F Fig). Taken together, these data indicate that T cells that are largely proficient for ATMIN and NBS1 are causative of the inflammatory phenotype.

Since regulatory T cells (T_reg_) are known to maintain T cell homeostasis we assessed their abundance by staining cells isolated from the spleens of control, ATMIN^ΔL^, NBS1^ΔL^ and ATMIN^ΔL^NBS1^ΔL^ mice for CD4 and Foxp3. We did not find a reduction in the proportion of Foxp3^+^ T_reg_ cells. The CD4^+^Foxp3^+^ cells were found to be slightly elevated in both ATMIN^ΔL^NBS1^ΔL^ and NBS1^ΔL^ mice (S8A and S8B Fig). Therefore we conclude that a decrease in Foxp3^+^ T_reg_ cells is not the cause for the auto activation of T cells that results due to a concomitant loss of ATMIN and NBS1, however, we cannot rule out that their function is impaired.

In summary, the ATMIN^ΔL^NBS1^ΔL^ mice (and to a lesser degree the NBS1^ΔL^ mice) display an immune activation phenotype on the periphery, coupled with T cell proliferation. The extent of the autoactivation is marginal in NBS1^ΔL^ mice but is exacerbated and deleterious upon simultaneous loss of ATMIN in T cells. Yet the proliferating and activated T cells were mostly ATMIN/NBS1 proficient.

### ATMIN and NBS1 are required to suppress DNA damage in T cells

**Fig 3:**
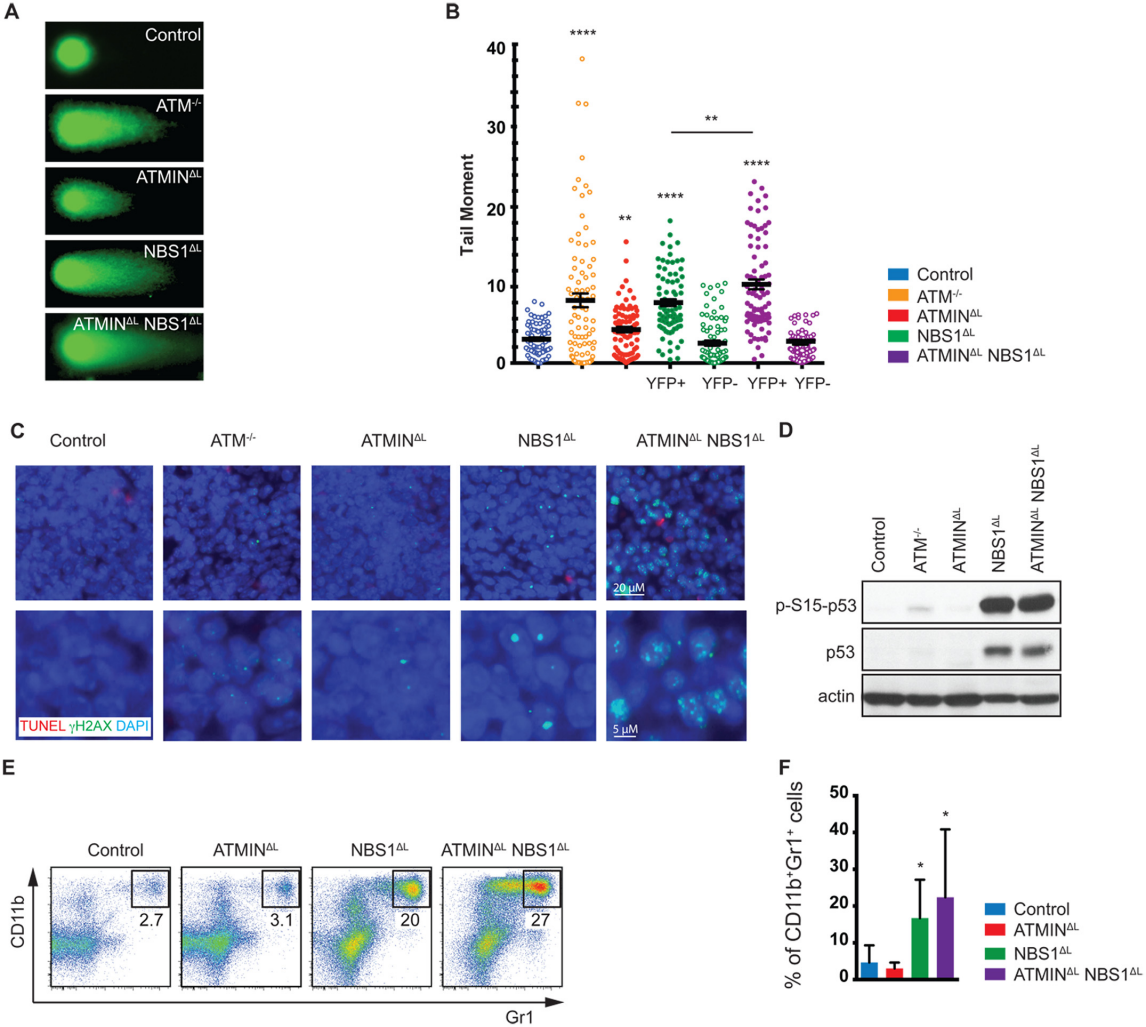
Loss of ATMIN and NBS1 in T cells leads to the accumulation of DNA damage. (A) Representative images of FACS sorted (for TCRβ^+^ populations) splenic T cells from control, ATM^-/-^, ATMIN^ΔL^, NBS1^ΔL^ and ATMIN^ΔL^NBS1^ΔL^ mice, analysed using the alkali comet assay. (B) Quantification of A. N = 3 mice per genotype. FACS sorted splenic T cells from NBS1^ΔL^ and ATMIN^ΔL^NBS1^ΔL^ mice were additionally sorted and analysed based on YFP expression. (C) Splenic sections from control, ATM^-/-^, ATMIN^ΔL^, NBS1^ΔL^ and ATMIN^ΔL^NBS1^ΔL^ mice were co-stained for TUNEL and γH2AX. Nuclei were counterstained with DAPI. (D) Western blot analysis of splenic cells from control, ATM^-/-^, ATMIN^ΔL^, NBS1^ΔL^ and ATMIN^ΔL^NBS1^ΔL^ mice for pS15-p53, total p53 and actin. (E) Representative flow cytometry data of CD11b^+^Gr1^+^ neutrophils in the spleen of control, ATMIN^ΔL^, NBS1^ΔL^ and ATMIN^ΔL^NBS1^ΔL^ mice. (F) Quantification of E. N = 7–10 mice per genotype. Error bars represent SEM (*P<0.05, **P<0.01, **** *P*<0.0001).

To test whether elevated DNA damage could be a contributing factor to T cell activation, we measured the presence of alkali-labile sites as well as DNA single- and double-strand breaks in splenic T cells using the alkali comet assay. We observed a significant contribution of ATMIN to suppress these types of lesions, as well as of NBS1 (Fig 3A and 3B). Indeed, splenic T cells isolated from ATMIN^ΔL^NBS1^ΔL^ mice displayed an elevated amount of DNA lesions. DNA lesions were observed in YFP^+^ T cells but not YFP^-^ T cells. DNA damage was also confirmed by γH2AX staining, where increased foci were observed in the nuclei of spleens from ATMIN^ΔL^NBS1^ΔL^ mice (Fig 3C). We also observed elevated levels of phosphorylated p53 in the spleens of both NBS1^ΔL^ and ATMIN^ΔL^NBS1^ΔL^ mice (Fig 3D). Since p53 is stabilized upon phosphorylation, the total levels of p53 were also increased (Fig 3D). This data for NBS1 is in line with a previous report that show increased apoptosis in NBS1-deficient neuronal cells, which is dependent on p53 [43].

Having observed elevated DNA damage, activation, inflammation and proliferation in the spleens of ATMIN^ΔL^NBS1^ΔL^ mice we next asked whether this could lead to neutrophil infiltration hence we stained for CD11b and Gr1 to identify neutrophils. We detected an enrichment of neutrophils (but potentially also monocytic cells) in the spleens of NBS1^ΔL^ mice and ATMIN^ΔL^NBS1^ΔL^ mice (Fig 3E and 3F). The recruitment of neutrophils might be a consequence of the observed T cell activation [47].

### T cells from ATMIN^ΔL^NBS1^ΔL^ mice display increased cytokine production

**Fig 4:**
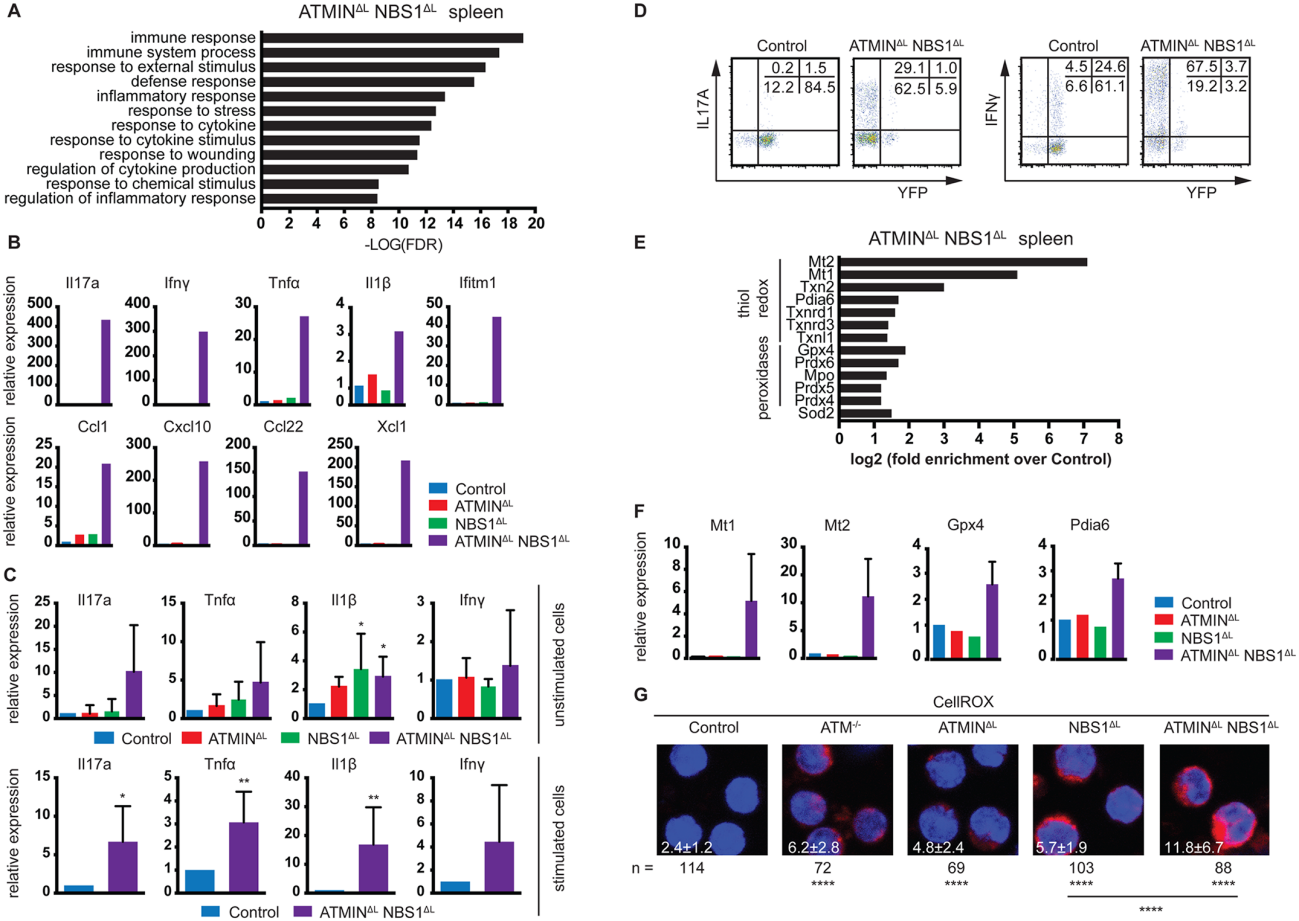
ATMIN^ΔL^NBS1^ΔL^ mice display enhanced cytokine and ROS production. (A) Gene ontology analysis of processes enriched in the spleen of ATMIN^ΔL^NBS1^ΔL^ mice identified by mRNA-sequencing. (B) Quantitative RT-PCR analysis of selected genes found to be enriched in A in the spleens of control, ATMIN^ΔL^, NBS1^ΔL^ and ATMIN^ΔL^NBS1^ΔL^ mice. Gene expression is normalized to mef1α control. (C) Quantitative RT-PCR analysis of expression of proinflammatory cytokines Il17a, Tnfα, Il1β and Ifnγ in splenic T cells of indicated mice with or without 12 hours *in vitro* stimulation with anti-CD3 and anti-CD28 antibodies. Gene expression is normalized to mef1α control. N = 4–8 mice per genotype. (D) Representative intracellular expression of IL17A and IFNγ assessed by flow cytometry, in YFP^-^ and YFP^+^ T (gated on the CD4^+^ population) cells in the spleen of control and ATMIN^ΔL^NBS1^ΔL^ mice after PMA and ionomycin stimulation. (E) Enrichment analysis of anti-oxidative genes in the spleen of ATMIN^ΔL^NBS1^ΔL^ mice identified by mRNA-sequencing. (F) Quantitative RT-PCR analysis of selected genes found to be enriched in E in the spleens of control, ATMIN^ΔL^, NBS1^ΔL^ and ATMIN^ΔL^NBS1^ΔL^ mice. Gene expression is normalized to mef1α control. (G) Splenic cells from control, ATM^-/-^, ATMIN^ΔL^, NBS1^ΔL^ and ATMIN^ΔL^NBS1^ΔL^ mice were stained with the CellROX reagent and counterstained with Hoechst 33258. Error bars represent SEM (*P<0.05, **P<0.01, ****P<0.0001).

To address the causes of the peripheral inflammation more closely, we performed expression analyses by RNA sequencing on total splenocytes isolated from control, ATMIN^ΔL^, NBS1^ΔL^ and ATMIN^ΔL^NBS1^ΔL^ mice. We chose to analyze total splenic RNA as we aimed to obtain a global representation of pathways and molecules affected in the spleens of moribund ATMIN^ΔL^NBS1^ΔL^ mice. Hence, we isolated RNA from spleens of moribund ATMIN^ΔL^NBS1^ΔL^ mice, along with ATMIN^ΔL^ and NBS1^ΔL^ mice. Compared to the ATMIN^ΔL^ and NBS1^ΔL^ mice, the moribund ATMIN^ΔL^NBS1^ΔL^ mice show a profound inflammatory phenotype (Fig 4A and S9A and S9B Fig). Among the most enriched gene ontology terms were ‘inflammatory response’ and ‘regulation of cytokine and chemokine production’. We selected several genes that were among those most highly upregulated in ATMIN^ΔL^NBS1^ΔL^ mice and validated their expression by quantitative RT-PCR analysis. We observed a dramatic increase in the expression of several proinflammatory markers such as Il17a, Ifnγ, Tnfα, IL-1β and Ifitm1, and chemokines such as Ccl1, Cxcl10, Ccl22 and Xcl1, in ATMIN^ΔL^NBS1^ΔL^ mice displaying splenomegaly, compared to the other genotypes (Fig 4B).

Since we observed that only a proportion of ATMIN^ΔL^NBS1^ΔL^ mice develop splenomegaly, we assessed the activation potential of T cells in the spleens of ATMIN^ΔL^NBS1^ΔL^ mice displaying no signs of splenomegaly. To this end we MACS sorted T cells to 90% purity and quantified the expression of a panel of cytokines in the presence or absence of *in vitro* stimulation (using anti-CD3 and CD28 antibodies). We detected increased levels of mainly Th1 and Th17 proinflammatory cytokines such as Il1β, Tnfα and Il17a specifically in splenic T cells from ATMIN^ΔL^NBS1^ΔL^ mice (Fig 4C). After *in vitro* stimulation there was a substantial increase in production of all displayed cytokines in ATMIN^ΔL^NBS1^ΔL^ mice. We confirmed these results by flow cytometry, showing that splenic T cells from ATMIN^ΔL^NBS1^ΔL^ mice produced high amounts of IL17A and IFNγ and similarly to previous findings, most of the cytokine producing cells were YFP^-^ (Fig 4D). These data indicate that in ATMIN^ΔL^NBS1^ΔL^ mice the expanded ATMIN/NBS1-proficient T cells are highly prone to eliciting an inflammatory response when stimulated *in vitro*, although the double-deficient mice do not show signs of splenomegaly.

### Splenocytes from ATMIN^ΔL^NBS1^ΔL^ mice display increased reactive oxygen species

Enlarged spleens from ATMIN^ΔL^NBS1^ΔL^ mice contain activated, proliferating ATMIN/NBS1-proficient T cells as well as neutrophils. Since neutrophils are known to produce reactive oxygen species (ROS), we investigated the expression levels of genes involved in the oxidative stress response from the RNA sequencing data obtained from ATMIN^ΔL^NBS1^ΔL^ mice (Fig 4E). We observed an increase in the expression of genes involved in the clearance of oxidative stress (Fig 4E). To validate this finding, we analysed the expression of selected genes involved in the detoxification of oxidative stress (Mt1, Mt2, Gpx4 and Pdia6) and confirmed their upregulation in the spleens of ATMIN^ΔL^NBS1^ΔL^ mice (Fig 4F). In support of these findings, we measured ROS production in splenocytes and showed that ROS production was increased in all genotypes but additively so in ATMIN^ΔL^NBS1^ΔL^ mice (Fig 4G).

### Loss of ATMIN and NBS1 in T cells leads to intestinal inflammation

**Fig 5:**
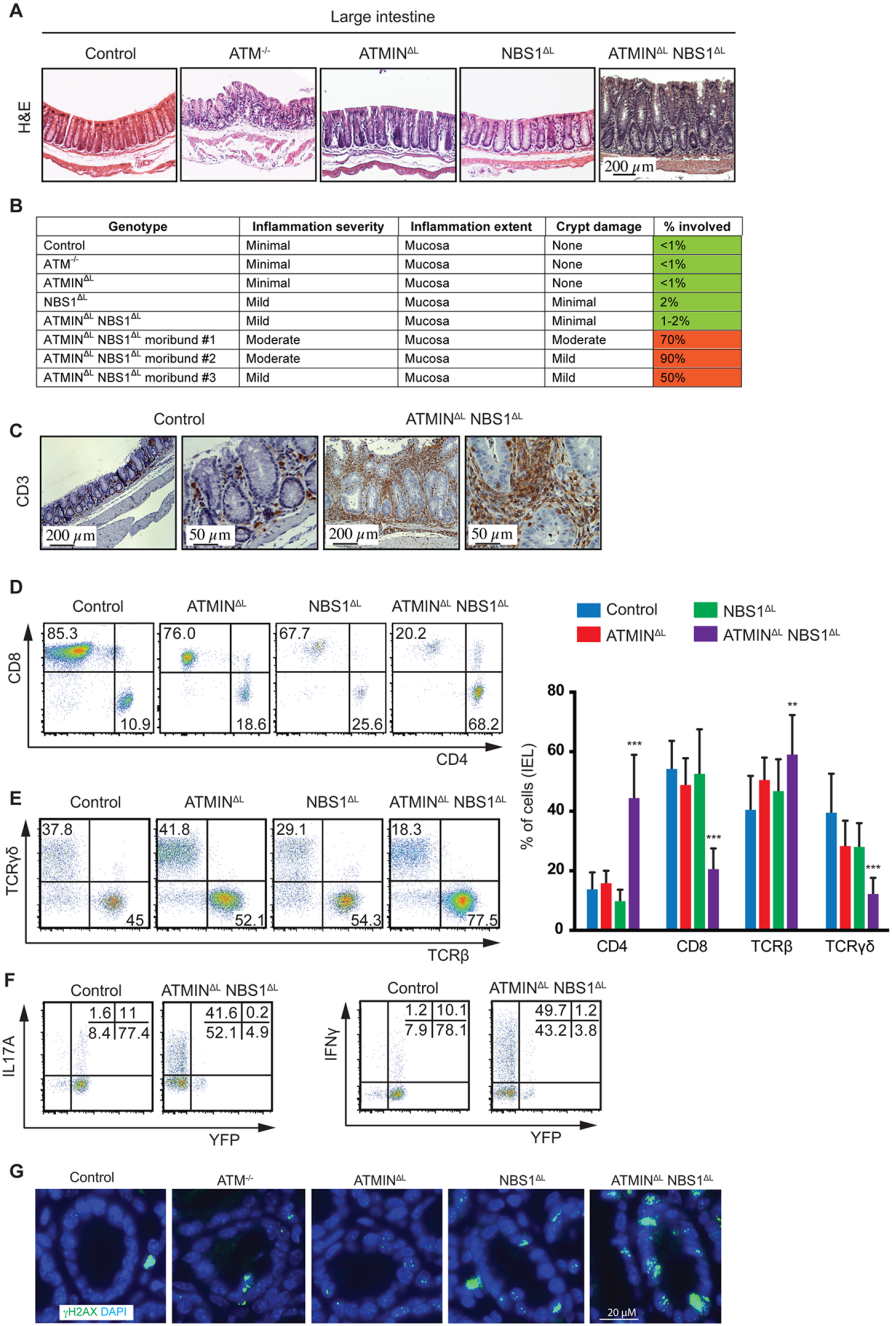
Loss of ATMIN and NBS1 leads to intestinal inflammation due to infiltration of cytokine-producing T cells. (A) Histological analysis by H&E staining of large intestine of control, ATM^-/-^, ATMIN^ΔL^, NBS1^ΔL^, ATMIN^ΔL^NBS1^ΔL^ mice at 12 weeks of age. (B) Histological scores of the large intestine of control, ATM^-/-^, ATMIN^ΔL^, NBS1^ΔL^, ATMIN^ΔL^NBS1^ΔL^ and 3 individual moribund ATMIN^ΔL^NBS1^ΔL^ mice. (C) Histological analysis by anti-CD3 staining of the large intestine of control and a moribund ATMIN^ΔL^NBS1^ΔL^ mouse. (D) Representative flow cytometry data of CD4 and CD8 expression, as well as (E) TCRβ and TCRγδ expression on isolated IELs from the small intestine of control, ATMIN^ΔL^, NBS1^ΔL^ and ATMIN^ΔL^NBS1^ΔL^ mice, along with the quantification of D-E. N = 5–8 mice per genotype. (F) Representative flow cytometry data of IL17A and IFNγ production by YFP^-^ and YFP^+^ IELs (gated on the CD4^+^ population) isolated from the small intestine of control and ATMIN^ΔL^NBS1^ΔL^ mice after PMA and ionomycin stimulation. (G) Large intestinal sections from control, ATM^-/-^, ATMIN^ΔL^, NBS1^ΔL^ and ATMIN^ΔL^NBS1^ΔL^ mice were stained for γH2AX and DAPI. Error bars represent SEM (**P<0.01, ***P<0.001).

A proportion of ATMIN^ΔL^NBS1^ΔL^ mice became moribund and displayed systemic inflammation, which also involved the intestine since we observed the development of spontaneous intestinal prolapses. We histologically investigated the large intestine that was found to be thickened and inflamed (Fig 5A). Moreover, histological scoring of the spontaneously sick ATMIN^ΔL^NBS1^ΔL^ mice revealed extensive inflammation of the intestine (Fig 5B).

Since we observed an infiltration of CD3^+^ T cells to the large intestine (Fig 5C), we next aimed to address what type of T cells these represented and whether the T cells were deficient or proficient for ATMIN/NBS1. Hence, we isolated intra-epithelial lymphocytes (IEL) from the small intestine of all genotypes, which revealed a specific enrichment of CD4^+^ T cells in ATMIN^ΔL^NBS1^ΔL^ mice (Fig 5D and 5E). The CD8^+^ T cell compartment was reduced accordingly. Similarly the TCRβ^+^ T cells were increased accompanied by a concomitant reduction in TCRγδ^+^ T cells; a population that is normally abundant in the intestine. In conclusion, we observed a severe inflammatory phenotype in ATMIN^ΔL^NBS1^ΔL^ mice characterized by infiltration of CD4^+^ T cells and a reduction of CD8^+^ T cells in the intestine.

In line with our observations from splenic T cells from ATMIN^ΔL^NBS1^ΔL^ mice, we observed increased amounts of IL17A and IFNγ in the IELs isolated from ATMIN^ΔL^NBS1^ΔL^ mice, which were mostly produced by YFP^-^ T cells (Fig 5F). Moreover, we observed elevated levels of γH2AX, indicative of DNA damage, in the intestine of ATMIN^ΔL^NBS1^ΔL^ mice (Fig 5G and S10 Fig). Hence, we conclude that in the intestine of ATMIN^ΔL^NBS1^ΔL^ mice, T cells proficient for ATMIN and NBS1 produce enhanced proinflammatory cytokines and interleukins, which drive severe inflammation.

### The IBD phenotype in ATMIN^ΔL^NBS1^ΔL^ mice is T cell mediated and transplantable

**Fig 6:**
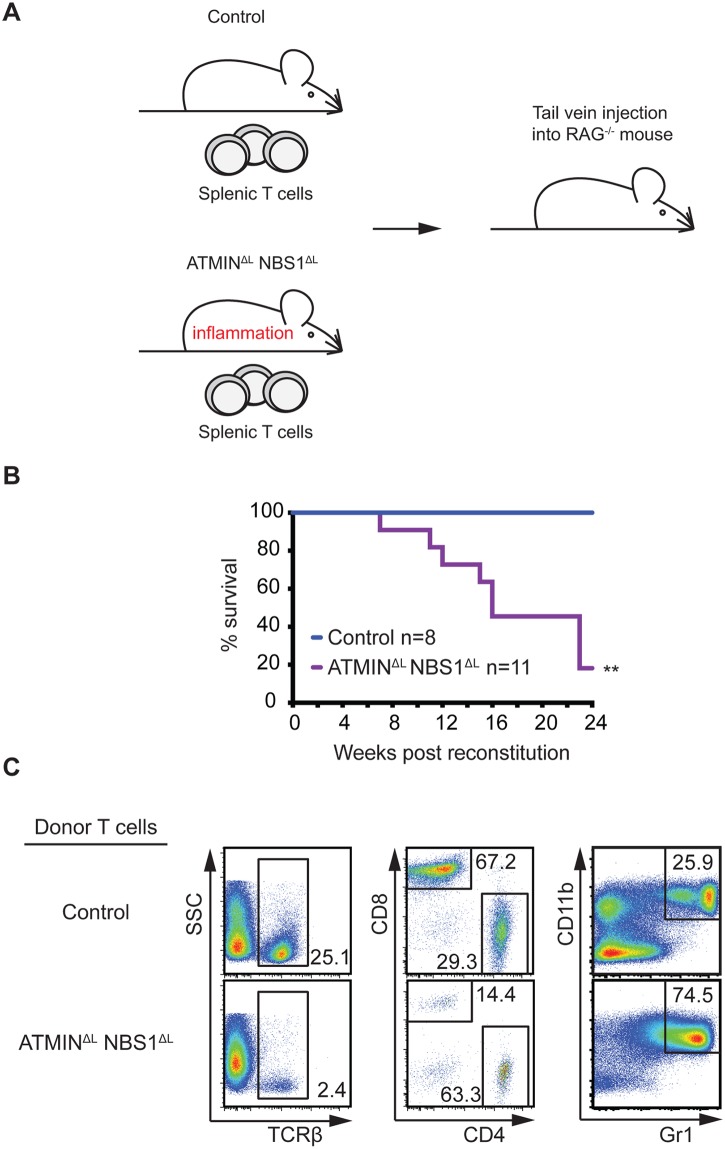
Systemic inflammation in ATMIN^ΔL^NBS1^ΔL^ mice is T cell intrinsic. (A) Model for the reconstitution of RAG^-/-^ mice reconstituted with intravenously injected with CD3^+^ TCRβ^+^ sorted splenic T cells from control or ATMIN^ΔL^NBS1^ΔL^ mice. (B) Kaplan-Meier survival curve of RAG^-/-^ mice that were reconstituted as in A. Survival was monitored for 24 weeks post reconstitution. (C) Representative flow cytometry plots of TCRβ^+^ T cells as well as CD4^+^ and CD8^+^ T cells (gated on the TCRβ^+^ population) and Gr1^+^CD11^+^ neutrophils in the spleen of RAG^-/-^ mice injected with control or ATMIN^ΔL^NBS1^ΔL^ T cells. **P<0.01.

To confirm that T cells are the driving cause of the severe inflammation in ATMIN^ΔL^NBS1^ΔL^ mice, we isolated CD3^+^TCRβ^+^ splenic T cells from control and ATMIN^ΔL^NBS1^ΔL^ mice and transferred these cells into immunodeficient RAG2^-/-^ mice (Fig 6A). RAG2^-/-^ mice injected with T cells from ATMIN^ΔL^NBS1^ΔL^ mice displayed high mortality due to severe inflammation, including that of the spleen and intestine (Fig 6B). Phenotypic characterization of splenic cells from moribund RAG2^-/-^ mice revealed a decreased proportion of TCRβ^+^ T cells along with an elevated percentage of CD4^+^ T cells (Fig 6C) hence recapitulating the phenotype of ATMIN^ΔL^NBS1^ΔL^ mice. We also detected an increase in CD11b^+^Gr1^+^ neutrophils in the spleens of the RAG2^-/-^ mice reconstituted with ATMIN^ΔL^NBS1^ΔL^ T cells (Fig 6C). Genotyping PCR was used to quantify the deletion status of ATMIN and NBS1 in T cells from host ATMIN^ΔL^NBS1^ΔL^ mice transferred to recipient RAG2^-/-^ mice 6 months post transfer. The deletion efficiency was found to be approximately 10–30% (S11A Fig), indicating that similar to the phenotype observed in ATMIN^ΔL^NBS1^ΔL^ mice, the activation of ATMIN/NBS1 proficient T cells leads to the intestine inflammation phenotype in this RAG2 transfer model. However, since the amount of deletion of ATMIN and NBS1 does not change over a 6-month engraftment period (i.e. 20–40% of the deletion efficiency prior to transfer, S11B Fig), it might be possible that ATMIN/NBS1 double-deficient T cells are also involved in the maintenance of the disease (in addition to its initiation) under this experimental setting. Taken together, these results clearly demonstrated that the inflammation phenotype observed in ATMIN^ΔL^NBS1^ΔL^ mice is due to defects in T cells (and not in B cells).

### Mice deficient for ATMIN and NBS1 in T cells produce inflammatory cytokines in the intestine and are hypersensitive to induction of colitis

**Fig 7:**
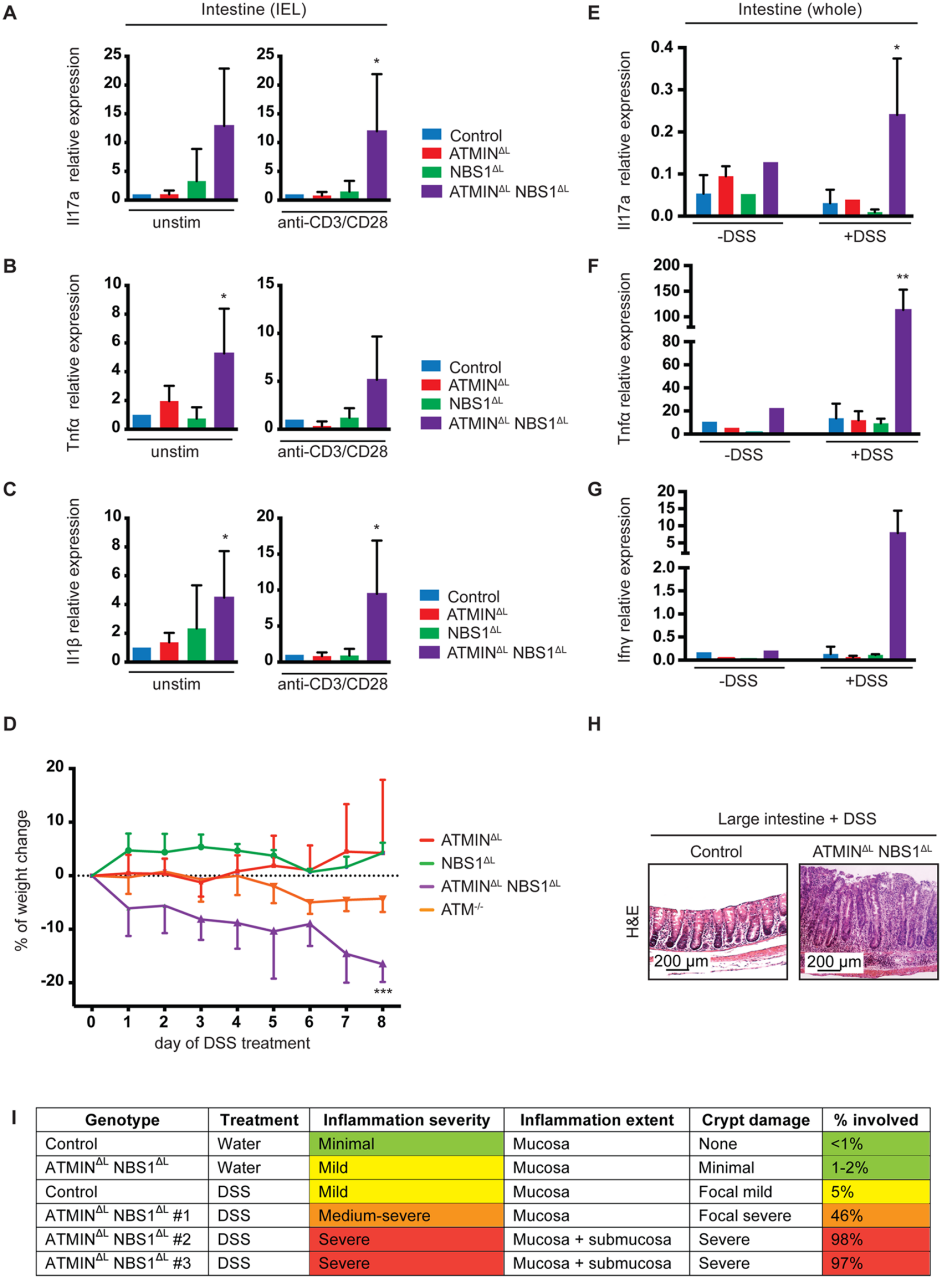
Mice deficient for ATMIN and NBS1 in T cells produce inflammatory cytokines in the intestine and are hypersensitive to colitis. (A-C) Quantitative RT-PCR analysis of expression of proinflammatory cytokines Il17a, Tnfα and Il1β in the IELs of control, ATMIN^ΔL^, NBS1^ΔL^ and ATMIN^ΔL^NBS1^ΔL^ with (‘anti-CD3/CD28’) or without (‘unstim’) 12 hour *in vitro* stimulation with anti-CD3 and anti-CD28 antibodies. Gene expression is normalized to mef1α control. N = 4–8 mice per genotype. (D) Percentage of weight change upon DSS treatment of ATMIN^ΔL^, NBS1^ΔL^, ATMIN^ΔL^NBS1^ΔL^ and ATM^-/-^ mice for 8 days. Each genotype is normalized to its respective control. N = 4 mice per genotype. (E-G) Quantitative RT-PCR analysis of expression of proinflammatory cytokines Il17a, Tnfα and Ifnγ in small intestine of DSS treated (‘+DSS’) or untreated (‘-DSS’) control, ATMIN^ΔL^, NBS1^ΔL^ and ATMIN^ΔL^NBS1^ΔL^ mice. N = 4 mice per genotype. (H) Histological analysis by H&E staining of large intestine of DSS treated control and ATMIN^ΔL^NBS1^ΔL^ mice. (I) Histological analysis of control and ATMIN^ΔL^NBS1^ΔL^ mice without (water) or with DSS. Error bars represent SEM (*P<0.05, **P<0.01, ***P<0.001).

Having observed increased cytokine production by IELs from ATMIN^ΔL^NBS1^ΔL^ mice, we next isolated IELs from control, ATMIN^ΔL^, NBS1^ΔL^ and ATMIN^ΔL^NBS1^ΔL^ mice and analyzed the expression of proinflammatory cytokines by quantitative RT-PCR. We assessed gene expression in both unstimulated and anti-CD3 and CD28 antibody stimulated IELs. We observed high expression levels of the inflammatory cytokines Il-1β, Tnfα and Il17a in both unstimulated and stimulated IEL from ATMIN^ΔL^NBS1^ΔL^ mice (Fig 7A–7C).

Since only a proportion of ATMIN^ΔL^NBS1^ΔL^ mice develop spontaneous colitis we assessed whether these mice would be more susceptible to chemically induced colitis with dextran sodium sulphate (DSS). The DSS model of colitis has similarities to human IBD hence is an ideal model to mimic this disease [48]. We treated control, ATMIN^ΔL^, NBS1^ΔL^, ATMIN^ΔL^NBS1^ΔL^ and ATM^-/-^ mice for 8 days with 2% DSS, at which point the control mice did not show any signs of weight loss. In striking contrast to ATMIN^ΔL^ and NBS1^ΔL^, only the ATMIN^ΔL^NBS1^ΔL^ mice were sensitive to DSS-induced colitis as apparent by the weight loss of approximately 20% in these mice over an 8-day period (Fig 7D). Our data also confirmed the reported mild sensitivity of ATM^-/-^ to DSS induced colitis [49].

Following DSS treatment, mice were sacrificed and the expression of inflammatory cytokines was assessed in the large intestine, as this is the tissue mostly affected following exposure to DSS. We detected a substantial increase in the expression of the inflammatory cytokines Il17a, Ifnγ and Tnfα after DSS treatment, specifically in ATMIN^ΔL^NBS1^ΔL^ mice (Fig 7E–7G). Moreover, we observed a thickening of the large intestine, in the DSS treated ATMIN^ΔL^NBS1^ΔL^ mice as assessed by histological analysis (Fig 7H). Assessment of the colitis score showed a severe inflammation of the intestine of ATMIN^ΔL^NBS1^ΔL^ mice treated with DSS (Fig 7I). These data indicate that although a proportion of ATMIN^ΔL^NBS1^ΔL^ mice develop spontaneous inflammation and colitis, these mice are prone to chemically induced colitis.

## Discussion

By utilizing murine models for the conditional deletion of ATMIN and/or NBS1 in T cells, achieved via the use of CD2-cre, we have identified a novel role for NBS1 in TCRα recombination. In this study we have confirmed findings showing that ATM is required for recombination of the TCRα locus [46] and our data indicate that this process is regulated by NBS1. Hence, NBS1 appears to be the cofactor of ATM that drives TCRα recombination. Loss of ATMIN does not affect TCRα recombination and furthermore loss of ATMIN in NBS1-deficient mice does not rescue this NBS1 dependent defect.

As well as uncovering a role for NBS1 in TCRα recombination, we show that loss of NBS1 leads to a block in T cell development at the DP stage of development. This novel finding differs from the developmental block reported by Saidi and colleagues [25] where the use of Lck-cre to mediate NBS1 deletion resulted in a T cell developmental block at the DN3 to DN4 stage [25]. The differences between the developmental block at DP and DN3-DN4 observed by Saidi and colleagues [25] could be due to the use of different cre lines, which delete with varying efficiency during T cell development, with CD2-cre appearing to delete target genes more efficiently. As with our findings for TCRα recombination, ATMIN does not play a role in T cell development and moreover loss of ATMIN cannot rescue NBS1-mediated functions with regard to T cell development. The inability of ATMIN-loss to rescue the NBS1-dependent reduction in thymic cellularity was unexpected as it contrasts to other cellular systems where loss of ATMIN rescues NBS1-dependent cellular lethality [35].

Unexpectedly, however, the combined loss of ATMIN and NBS1 results in spontaneous activation of peripheral T cells, including in the spleen and intestine that results in the development of intestinal prolapses in approximately 30% of ATMIN^ΔL^NBS1^ΔL^ mice. Moreover, in an *in vivo* experimental system for intestinal colitis, using DSS to mimic IBD, we report enhanced colitis, significantly and specifically, in ATMIN^ΔL^NBS1^ΔL^ mice. Although we observe a tendency towards spontaneous T cell activation in mice lacking only NBS1, as indicated by increased antigen experienced cells in the spleen and increased proliferation of splenic T cells, this phenotype is not pronounced enough to give rise to pathology, that is spontaneous colitis development. Hence, in T cells, loss of ATMIN exacerbates a phenotype of spontaneous T cell activation observed upon loss of NBS1. Interestingly, the T cells that are activated in the ATMIN^ΔL^NBS1^ΔL^ mouse model are predominantly T cells that have ‘escaped’ cre-mediated deletion.

One could speculate that the spontaneous inflammation driven by ATMIN/NBS1-proficient T cells is a secondary phenotype that occurs due to lymphopenia. In such a scenario the wild-type T cells in ATMIN^ΔL^NBS1^ΔL^ mice proliferate to fill an ‘empty space’. Yet we would argue against this since we do not observe spontaneous intestinal inflammation in NBS1^ΔL^ mice that are as lymphopenic as ATMIN^ΔL^NBS1^ΔL^ mice. Furthermore, spontaneous inflammation is not a general feature of lymphopenic mice and to our knowledge this is the first report of a DNA repair deficiency that leads to spontaneous colitis.

ATM-deficient mice do not develop spontaneous systemic inflammation and yet the combinatorial loss of ATMIN and NBS1 does. There are two potential explanations for this; firstly, these cofactors have ATM-independent roles that contribute to the development of colitis. Secondly, when removing these two cofactors, ATM is still present but it cannot function. This would allow the kinase to function in a ‘dominant-negative manner’, binding its substrates but being unable to phosphorylate them. In doing so, other kinases belonging to the ATM-superfamily (such as DNA-PKcs) would be unable to compensate for ATM activity. In support of this, the ATM-deficient mouse is viable whereas the kinase-dead ATM mouse is lethal [3,50,51].

**Fig 8:**
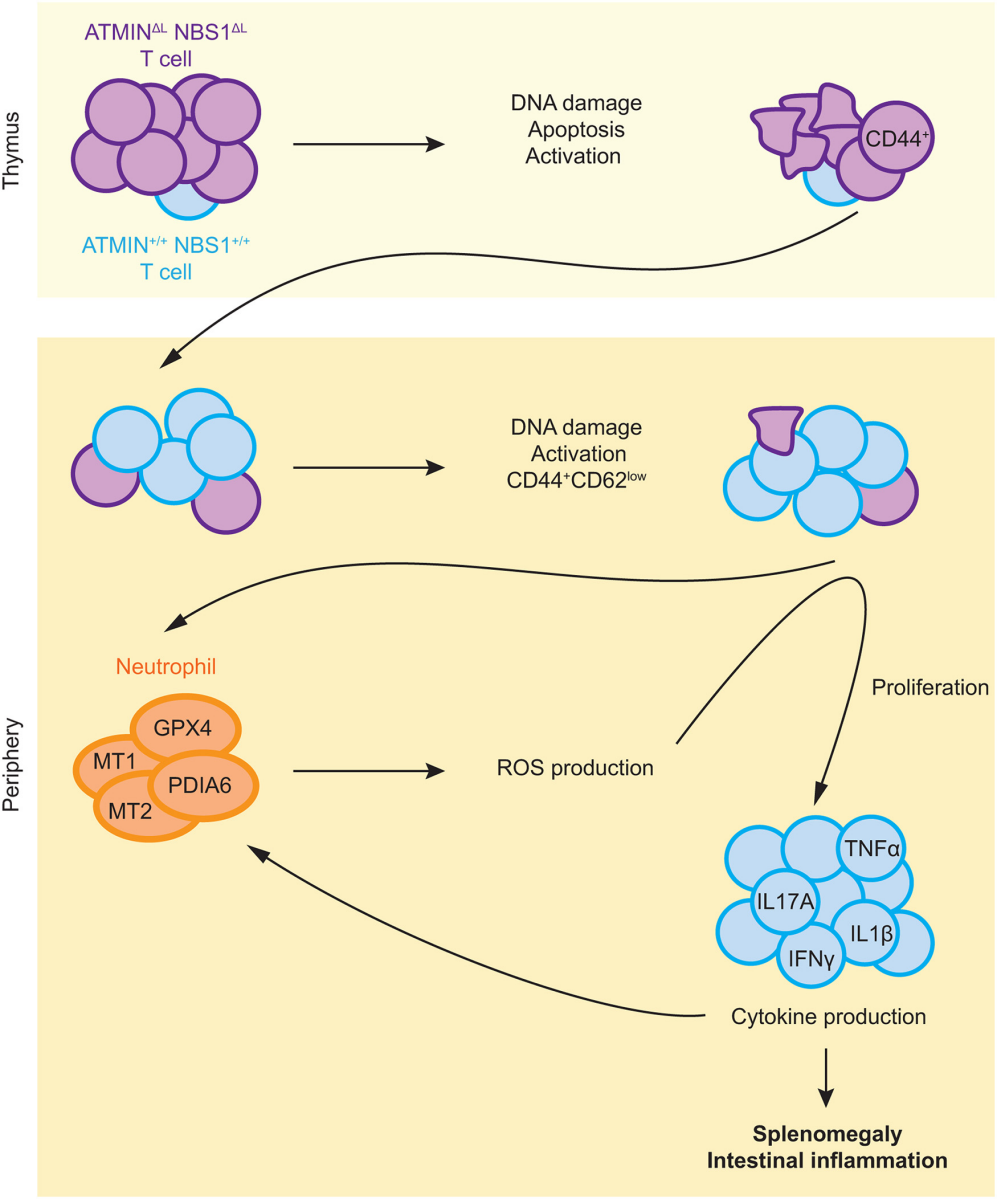
Model for the role of ATMIN and NBS1 in suppressing T cell activation and inflammation. Proposed model depicting mechanisms of inflammation caused by concomitant deletion of ATMIN and NBS1 (details can be found in the text).

We consolidate the data presented in this manuscript in the form of a model as displayed in Fig 8. Co-deletion of ATMIN and NBS1 in T cells leads to excessive DNA damage, and in turn, increased apoptosis in T cells, leading to a reduction in T cells numbers. Surviving, mostly wild-type, T cells move to the periphery where they show increased proliferation and activation, as marked by the production of cytokines, including IL-17A. Subsequently, neutrophil infiltration leads to ROS production hence explaining the increased expression of ROS-detoxifying genes, including Mt1, Mt2, Gpx4 and Pdia6 that we observe in the spleens of ATMIN^ΔL^NBS1^ΔL^ mice. The increased ROS could also exacerbate the DNA damage observed in ATMIN^ΔL^NBS1^ΔL^ cells. We propose that the increase in neutrophils and proliferating T cells in moribund ATMIN^ΔL^NBS1^ΔL^ mice are the causes of splenomegaly and intestinal inflammation that eventually leads to premature death of ATMIN^ΔL^NBS1^ΔL^ mice. Hence our data support a model where ATMIN and NBS1 proficient T cells are the source of inflammation.

In summary we have generated a novel mutant mouse strain that develops an IBD-like phenotype that occurs due to the combined loss of the ATM cofactors, ATMIN and NBS1 in T cells. The underlying genetic causes of many patients displaying immunodeficiency and/or IBD are to a large extent unknown. Here we shed light on factors leading to the development of such defects.

## Materials and Methods

### Ethics statement

Mice were maintained and bred at the Institute of Molecular Biotechnology, Vienna. All experimental procedures were approved by the ethical committee of the Medical University of Vienna and by Federal Ministry of Science and Research and conform to Austrian law (license number: BMWF-66.009/0069-II/3b/2012).

### Mice

The generation of ATMIN^F/F^ and NBS1^F/F^ mice has been described previously [33,43]. ATMIN^F/F^ and NBS1^F/F^ mice were bred to achieve ATMIN^F/F^NBS1^F/F^ mice. For cre-mediated deletion ATMIN^F/F^, NBS1^F/F^ and ATMIN^F/F^NBS1^F/F^ mice were crossed with heterozygous CD2-cre mice [44] and were designated ATMIN^ΔL^, NBS1^ΔL^ and ATMIN^ΔL^NBS1^ΔL^. ATM mice were bred as ATM^+/-^ as described previously [3]. Control mice include ATMIN^F/F^-CD2cre^-^, NBS1^F/F^-CD2cre^-^, ATMIN^F/F^NBS1^F/F^-CD2cre^-^ and CD2cre^+^ mice. Unless otherwise stated, all mice were used at 6–12 weeks of age. CD2-cre deletion efficiency and genotyping of mice were determined on DNA using PCR-based assay. Primers are listed in Table 1. RAG2^-/-^ mice were used for the T cell reconstitution experiments [52].

**Table 1:** List of PCR primers used in this study for genotyping, Southern blotting and for analysis of cytokine production by quantitative RT-PCR.

Application	Primer name	Primer sequence 5’– 3’
Genotyping	ATMIN F	TCAGCATCTTCTCCAGAGAGACAG
	ATMIN R	CACATGTGTACAGCACATTCATTG
	ATMIN delta	CTCAGGGTACACATACTATGCTTGC
	NBS1 F	CAGGGCGACATGAAAGAAAAC
	NBS1 R	AATACAGTGACTCCTGGAGG
	NBS1 delta	ATAAGACAGTCACCACTGCG
	ATM WT F	GCTGCCATACTTGATCCATG
	ATM WT R	TCCGAATTTGCAGGAGTTG
	ATM mut F	CTTGGGTGGAGAGGCTATTC
	ATM mut R	AGGTGAGATGACAGGAGATC
	CD2-cre F	AGATGCCAGGACATCAGGAACCTG
	CD2-cre R	ATCAGCCACACCAGACACAGAGATC
Southern blotting	Jβ2	TGAGAGCTGTCTCCTACTATCGATT
	Vβ5.1	GTCCAACAGTTTGATGACTATCAC
	Vβ8.2	CCTCATTCTGGAGTTGGCTACCC
	Thy1 F	CCATCCAGCATGAGTTCAGC
	Thy1 R	GCATCCAGGATGTGTTCTGA
	Dβ2	GTAGGCACCTGTGGGGAAGAAACT
Quantitative RT-PCR	mEF1α F	GCAAAAACGACCCACCAATG
	mEF1α R	GGCCTGGATGGTTCAGGATA
	IFNγ F	TTCAAGACTTCAAAGAGTCTGAGG
	IFNγ R	ATCTGGAGGAACTGGCAAAA
	TNFα F	ACCGTCAGCCGATTTGCTAT
	TNFα R	TTGACGGCAGAGAGGAGGTT
	IL6 F	ACCACGGCCTTCCCTACTTC
	IL6 R	TTGGGAGTGGTATCCTCTGTGA
	IL17A F	CCTGGCGGCTACAGTGAAG
	IL17A R	TTTGGACACGCTGAGCTTTG
	Xcl1 F	GGCCATGAGAGCTGTAATTTTTG
	Xcl1 R	TGGCTTCTGGATCAGCACAA
	Ccl1 F	TGATCCCCCAGCTGTGGTA
	Ccl1 R	GTTGAGGCGCAGCTTTCTCTA
	Cxcl10 F	GATGACGGGCCAGTGAGAA
	Cxcl10 R	GCTCGCAGGGATGATTTCAA
	Ccl22 F	TGGTGGCTCTCGTCCTTCTT
	Ccl22 R	ACCATAGGGACCTGCATCAGA
	Ifitm1 F	GCAGCAAGAGGTGGTTGTACTG
	Ifitm1 R	TGGTGGCTGTCGCAGAAG

### *In vivo* BrdU incorporation

For detection of cell proliferation, mice were injected with 1 mg BrdU or supplemented with 0.8 mg/ml of BrdU in drinking water and analysed at the indicated time points.

### Colitis induction

For colitis induction, mice were challenged with 2% (mass/vol) dextran sodium sulphate (DSS; molecular weight 36–50 kDa; MP Biomedicals) in autoclaved drinking water ad libitum for 8 consecutive days. Weight of the animals was monitored every day. At the end of the treatment mice were sacrificed and the colon tissue was analyzed for cytokine production by quantitative reverse transcription (RT)-PCR.

### T cell reconstitution

RAG2^-/-^ mice were injected intravenously with 5x10^5^ of sorted splenic CD3^+^ TCRβ^+^ T cells in 200 μl of PBS. Moribund mice were sacrificed and samples were analyzed by flow cytometry.

### Histology

Tissue was fixed directly after harvesting in 4% paraformaldehyde solution and transferred into 70% ethanol after 24 hours. The samples were dehydrated using an increasing ethanol series and embedded in paraffin. Tissue sections were prepared using a microtome at a thickness of 5 μm. Samples were then rehydrated using xylene, ethanol solutions and water. Hematoxylin and eosin (H&E) staining and CD3 (Dako) staining was performed and finally slides were mounted in Entellan (Merck) and subjected to microscopy. Axio Imager A1 microscope (Zeiss) and Axio Cam MRc5 were used to acquire the images. Alternatively, the rehydrated samples were stained with an anti-γH2AX antibody (Cell Signaling) and with an In Situ Cell Death Detection Kit (Roche), according to manufacturers’ instructions. Samples were counterstained with diamidino-2-phenylindole (DAPI). Images were acquired on an Axio Imager M2 microscope (Zeiss).

### Comet assay

Cells at a density of 5×10^4^ were washed in pre-chilled PBS and then mixed in 100 μL 0.6% low melting agarose (Sigma, type VII) maintained at 37°C. The cell suspension was then immediately layered onto pre-chilled frosted glass slides pre-coated with 1.5% agarose and maintained in the dark at 4°C for all following steps. Slides were immersed in pre-chilled lysis buffer (2.5 M NaCl, 10 mM Tris–HCl, 100 mM EDTA pH 8.0, 1% Triton X-100, 1% DMSO, pH 10; DMSO and Triton X added shortly before use) overnight. Slides were washed with pre-chilled distilled water (2×10 minutes), and next placed for 45 minutes in pre-chilled alkaline electrophoresis buffer (55 mM NaOH, 1 mM EDTA, 1% DMSO). Electrophoresis was conducted at 30 V for 25 minutes, followed by neutralisation in 400 mM Tris–HCl pH 7.0 for 1 h. Finally, DNA was stained with SYBR Gold (1:10,000 dilution in H_2_O; Life Technologies) for 10 minutes. The comet tail moment was measured for at least 50 cells per sample in 3 replicates using the CASP image-analysis program [53].

### *In vitro* staining for oxidative stress

To detect ROS, splenic T cells were plated on a poly-L lysine (Sigma) coated plate (Corning) and stained with 5 μM CellROX Deep Red Reagent (Life Tech) for 30 minutes at 37°C, washed with PBS, fixed in 4% paraformaldehyde 10 minutes and counterstained with 5 μg/ml Hoechst 33258 for 5 min. Quantification of immunofluorescence images with CellROX was performed based on the mean fluorescence intensity of cytoplasmic area defined by the distance from the nuclei using the CellProfiler cell image analysis software v2.0 [54].

### Stimulation of splenic T cells

T cells were isolated from the spleen using the Pan T Cell Isolation Kit II (Miltenyi Biotec) according to manufacturer’s instructions. For cytokine production experiments, cells were stimulated with 25 ng/ml PMA and 10 mg/ml ionomycin in the presence of 10mg/ml Brefeldin A (all from Sigma) overnight. For assessment of cytokine gene expression by quantitative RT-PCR, cells were incubated in a 48-well plate, at 37°C with 5% CO_2_ and 3% O_2_ in the presence or absence of 2 μg/ml anti-CD3 and anti-CD28 immobilized antibodies (both from BD) overnight.

### Isolation of intraepithelial lymphocytes

Intraepithelial lymphocytes (IEL) were isolated from the small intestine. In brief, the small intestine was removed and flushed with PBS. The tissue was cut into pieces and incubated with RPMI medium containing 5 mM EDTA three times for 15 minutes Supernatant was collected and centrifuged and cells were purified on a Percoll (Sigma) gradient. Subsequently cells were subjected to flow cytometry analysis. In some cases, cells were cultured in RPMI (Invitrogen) supplemented with penicillin and streptomycin (Invitrogen), 10% FCS (Invitrogen) and mercaptoethanol. One x 10^5^ cells were incubated in a 96-well plate, at 37°C with 5% CO_2_ and 3% O_2_ in the presence or absence of 2 μg/ml anti-CD3 and anti-CD28 immobilized antibodies (both from BD) overnight. Cells were then harvested and used for quantitative RT-PCR analysis to determine cytokine expression. For cytokine production experiments, cells were stimulated with 25 ng/ml PMA and 10 mg/ml ionomycin in the presence of 10mg/ml Brefeldin A (all from Sigma) overnight.

### Flow cytometry

Cells were washed with PBS containing 0.5% BSA and incubated for 30 minutes on ice with the following antibodies: anti-CD4 (RM-4.5; eBioscience), anti-CD8 (53.6.7; BD), anti-CD44 (IM.7; BD), anti-CD25 (PC61; BD), anti-CD62L (MEL14; Biolegend), anti-TCRβ (H57-597; eBioscience), anti-TCRγδ (BD), anti-CD69 (H1.2F3; eBioscience), anti-CD11b (M1/70; BD), anti-Gr1 (RB6-8C5; eBioscience) and anti-HSA (M1/69, eBioscience). Cells were then washed in PBS with 0.5% BSA and data was collected using a Fortessa cytometer (BD Bioscience) and analyzed using FlowJo software (Treestar, Ashland, OR).

In the case of intracellular staining, cells were fixed and permeabilized using the Foxp3 buffer staining kit (eBioscience) according to the manufacturer’s instructions prior to staining for intracellular Foxp3 expression using an anti-Foxp3 antibody (FJK-16s, BD), for 30 minutes.

For Annexin V staining, cells were washed with PBS and stained with BD Pharmingen Annexin V Apoptosis Detection Kit I according to the manufacturer’s instructions.

The detection of BrdU was performed using BD Pharmingen BrdU Flow Kit according to the manufacturer’s instructions.

### Quantitative PCR

Splenocytes and IELs were harvested and RNA was isolated from cells using phenol-chlorophorm extraction. RNA was treated with 1 μl DNase (Sigma) and then reverse transcribed with the SuperScript III Reverse Transcriptase protocol (Invitrogen) to obtain cDNA. An amount of 50 ng of cDNA template was used for the qRT-PCR using SYBR Green qPCR Mastermix (Qiagen). For determination of cytokine expression mEF1α was used as reference gene. Alternatively, the DP population of thymocytes (CD4^+^CD8^+^) was isolated using fluorescence activated cell sorting (FACS). PCR quantification of TCR recombination regions was performed as published previously using total DNA from the isolated DP (CD4^+^CD8^+^) thymocytes [46]. The PCR was performed on a 7900HT Fast Real-Time PCR System (Applied Biosystems).

### Southern blotting for TCRβ recombination

The DN4 population (CD4^-^CD8^-^CD25^-^CD44^-^) of thymocytes was isolated using fluorescence activated cell sorting. Cells were lysed and subjected to PCR amplification of selected recombination regions using the following primers combinations: Jβ2 and Vβ5.1; Jβ2 and Vβ8.2; Thy1 F and Thy1 R for which the sequences are found in Table 1.

The Thy1 non-recombining region was used as a positive control. PCR products were separated on a 1.2% agarose gel and blotted onto a Hybond N^+^membrane and subjected to Southern blot analysis using a TCRβ probe which corresponds to the Jβ2.6 fragment and was obtained by PCR amplification with the Dβ2 and Jβ2 primers followed by gel purification. The Thy1 probe was generated by isolating the PCR fragment resulting from amplification using the Thy1 primers and gel purification. Both probes were labeled using the RandomPrimed DNA Labeling Kit (Roche Life Science) and α-^32^P-dCTP (Hartmann Analytic).

### Whole genome RNA-sequencing

The amount of total RNA was quantified using Qubit 2.0 Fluorometric Quantitation system (Life Technologies) and the RNA integrity number (RIN) was determined using Experion Automated Electrophoresis System (Bio-Rad). RNA-seq libraries were prepared with TruSeq Stranded mRNA LT sample preparation kit (Illumina) using Sciclone and Zephyr liquid handling robotics (PerkinElmer). Library amount was quantified using Qubit 2.0 Fluorometric Quantitation system (Life Technologies) and the size distribution was assessed using Experion Automated Electrophoresis System (Bio-Rad). For sequencing libraries were pooled, diluted and sequenced on Illumina HiSeq 2000 using 50 bp single-read chemistry. Base calls provided by the Illumina Realtime Analysis software were converted into BAM format using Illumina2bam and demultiplexed using BamIndexDecoder (https://github.com/wtsi-npg/illumina2bam). Transcriptome analysis was performed using the Tuxedo suite. TopHat2 (v2.0.10, http://genomebiology.com/2013/14/4/R36/abstract) was supplied with reads passing vendor quality filtering (PF reads) and the Ensembl transcript set (Mus musculus, e73, September 2013) as reference. TopHat2 analyses were run independently for each replicate. Cufflinks (v2.1.1, http://www.nature.com/nbt/journal/v31/n1/full/nbt.2450.html) was used to assemble transcripts from spliced read alignments, using the Ensembl e73 transcriptome as the reference as well as de novo assembly of transcript models. Differential expression was assessed with Cuffdiff v2.1.1 (http://www.nature.com/nbt/journal/v28/n5/full/nbt.1621.html). Transcriptome sets of all replicates for each sample group were combined with Cuffmerge. Finally, cummeRbund (http://www.bioconductor.org/packages/release/bioc/html/cummeRbund.html) and biomaRt (http://www.bioconductor.org/packages/release/bioc/html/biomaRt.html) were used in combination with custom R scripts to perform quality assessment and further refine analysis results.

### Western blotting

Cells were extracted in RIPA lysis buffer (NEB) supplemented with protease inhibitors (Sigma) and phosphatase inhibitors (Sigma, NEB). Western blots were performed using standard procedures. Protein samples were separated by SDS–PAGE (3–8% or 4–12% gradient gels; Invitrogen), and subsequently transferred onto nitrocellulose membranes. All primary antibodies were used at 1:1000 dilution and secondary antibodies at 1:5000. The following antibodies were used: ATM (Santa Cruz), ASCIZ (Millipore); p95 (known as NBS1) (NEB), β-actin (Sigma), pS15-p53 (Cell Signalling), pS824-KAP1 (Bethyl Labs), total p53 (Pab-421; CR-UK generated antibody) and HRP-conjugated goat anti-mouse or rabbit IgG (Sigma).

### Statistical analysis

The statistical significance of differences between the means of individual experimental groups compared to the control group was calculated using the Student’s t-test. Values with a p<0.05 were considered as statistically significant.

## Supporting Information

S1 Fig Deletion of ATMIN and NBS1 using CD2-cre. (A) Genotyping PCR for floxed, wild type (‘WT’) and deleted (‘Δ’) alleles of ATMIN and NBS1 performed on DNA from thymus or spleen samples. The PCR for CD2-cre was performed on DNA from tail. Western blot analysis of (B) thymi and (C) spleens from control, ATM^-/-^, ATMIN^ΔL^, NBS1^ΔL^ and ATMIN^ΔL^NBS1^ΔL^ mice probed for ATMIN, NBS1, ATM and actin.
(TIF)

S2 Fig Assessment of YFP expression in the thymus. (A) Representative flow cytometry data of YFP expression in DN, DP, SP CD4 and SP CD8 T cells in thymus of control, ATMIN^ΔL^, NBS1^ΔL^ and ATMIN^ΔL^NBS1^ΔL^ mice. (B) Representative flow cytometry data of YFP expression in DN1-4 T cells in thymus of control, ATMIN^ΔL^, NBS1^ΔL^ and ATMIN^ΔL^NBS1^ΔL^ mice. (C) Genotyping PCR for floxed, wild type (‘WT’) and deleted (‘Δ’) alleles of NBS1 performed on DNA from DN1-4 T cells from thymus of NBS1^ΔL^ mice.
(TIF)

S3 Fig NBS1 is required for T cell development. (A) Quantification of T cell subpopulations in the thymus measured by flow cytometry following staining for CD4, CD8, TCRβ and TCRγδ in control, ATMIN^ΔL^, NBS1^ΔL^ and ATMIN^ΔL^NBS1^ΔL^ mice. N = 4–8 mice per genotype. (B) Quantification of T cell subpopulations in the spleen measured by flow cytometry following staining for CD4, CD8, TCRβ and TCRγδ in control, ATMIN^ΔL^, NBS1^ΔL^ and ATMIN^ΔL^NBS1^ΔL^ mice. (C) Representative FACS plots of B. N = 3–5 mice per genotype. Error bars represent SEM (*P<0.05, **P<0.01, ***P<0.001).
(TIF)

S4 Fig ATMIN and NBS1 are dispensable for TCRβ recombination, survival and proliferation of DN thymic T cells. (A) Southern blot analysis of Vβ8.1-Jβ2 and Vβ5.1-Jβ2 recombination regions in FACS-sorted DN4 (CD25^-^CD44^-^) thymocytes of control, ATMIN^ΔL^, NBS1^ΔL^ and ATMIN^ΔL^NBS1^ΔL^ mice. Thy1 is used as a loading control. (B) Representative flow cytometry data of DN1 (CD25^-^CD44^+^), DN2 (CD25^+^CD44^+^), DN3 (CD25^+^CD44^-^) and DN4 (CD25^-^CD44^-^) T cell subpopulations in thymi of control, ATMIN^ΔL^, NBS1^ΔL^ and ATMIN^ΔL^NBS1^ΔL^ mice. N = 5–7 mice per genotype. (C) Quantification of B. (D) Representative flow cytometry data of Annexin V^+^ apoptotic T cells in DN (CD4^-^CD8^-^) population of thymi in mice indicated in A. (E) Quantification of d. N = 4 mice per genotype. (F) Representative flow cytometry results of BrdU^+^ DN T cells in thymus of mice indicated in A, assessed following 2 days of *in vivo* BrdU incorporation. (G) Quantification of F. N = 4 mice per genotype.
(TIF)

S5 Fig NBS1 is required for TCRα recombination, which is not affected by concomitant loss of ATMIN. (A) Schematic representation of the V to J recombination events in the TCRα locus. Arrows indicate forward and reverse primers used to amplify the selected V10 to J2 region. (B) Quantitative RT-PCR analysis of eight Vα10-Jα recombination regions in purified DP (CD4^+^CD8^+^) thymic T cells from control, ATMIN^ΔL^, NBS1^ΔL^, ATMIN^ΔL^NBS1^ΔL^ and ATM^-/-^ mice. Results are normalized to the control DP thymic T cells. N = 3 mice per genotype. Error bars represent SEM (*P<0.05).
(TIF)

S6 Fig Activation of CD4^+^ T cells occurs in the thymus of ATMIN^ΔL^NBS1^ΔL^ mice. (A) Representative flow cytometry data of CD44 expression on CD4^+^ and CD8^+^ thymocytes in control, ATMIN^ΔL^, NBS1^ΔL^ and ATMIN^ΔL^NBS1^ΔL^ mice. (B) Quantification of A.
(TIF)

S7 Fig YFP^+^ T cells are reduced in the spleen of ATMIN^ΔL^NBS1^ΔL^ mice. (A) Representative flow cytometry data of activated (CD62L^low^CD44^+^) CD4^+^ and CD8^+^ T cells in the spleen of control, ATMIN^ΔL^, NBS1^ΔL^ and ATMIN^ΔL^NBS1^ΔL^ mice. (B) Representative flow cytometry data of YFP^+^ T cells (gated on the TCRβ^+^ population) in the spleen of control, ATMIN^ΔL^, NBS1^ΔL^ and ATMIN^ΔL^NBS1^ΔL^ mice. (C) Representative flow cytometry data of YFP^+^ activated (CD44^+^CD62L^low^) T cells in the spleen of control, ATMIN^ΔL^, NBS1^ΔL^ and ATMIN^ΔL^NBS1^ΔL^ mice. (D) Representative flow cytometry data of YFP^+^ proliferating (BrdU^+^) T cells in the spleen of control, ATMIN^ΔL^, NBS1^ΔL^ and ATMIN^ΔL^NBS1^ΔL^ mice. (E) Genotyping PCR of control, two biological replicates of YFP^-^ ATMIN^ΔL^NBS1^ΔL^ and YFP^+^ ATMIN^ΔL^NBS1^ΔL^ FACS sorted (for TCRβ^+^ populations) splenic T cells, without TCR stimulation (‘naïve T cells’) or with TCR stimulation (‘activated T cells’). (F) Genotyping PCR of dilution series of control versus ATMIN^ΔL^NBS1^ΔL^ T cells from the thymus.
(TIF)

S8 Fig Regulatory T cells are not reduced in ATMIN^ΔL^NBS1^ΔL^ mice. (A) Representative flow cytometry data of CD4^+^Foxp3^+^ regulatory T (gated on the TCRβ^+^ population) cells in the spleen of control, ATMIN^ΔL^, NBS1^ΔL^, ATMIN^ΔL^NBS1^ΔL^ and ATM^-/-^ mice. N = 5–7 mice per genotype. (B) Quantification of A. Error bars represent SEM (*P<0.05, **P<0.01).
(TIF)

S9 Fig Expression profiling does not indicate an inflammatory phenotype in the spleens of ATMIN^ΔL^ or NBS1^ΔL^ mice. (A) Gene ontology analysis of processes enriched in spleen of ATMIN^ΔL^ mice identified by mRNA-sequencing. (B) Gene ontology analysis of processes enriched in spleen of NBS1^ΔL^ mice identified by mRNA-sequencing.
(TIF)

S10 Fig Intestine of ATMIN^ΔL^ or NBS1^ΔL^ mice displays infiltration of T cells and an accumulation of DNA damage. Intestines of control, ATMIN^ΔL^, NBS1^ΔL^ and ATMIN^ΔL^NBS1^ΔL^ mice were stained by H&E and for γH2AX as well as CD3.
(TIF)

S11 Fig Analysis of deletion status of ATMIN and NBS1 prior to and following engraftment into RAG^-/-^ mice. (A) Genotyping PCR for floxed and deleted (‘Δ’) alleles of ATMIN and NBS1 was performed on DNA from FACS sorted (TRCβ^+^ populations) T cells isolated from reconstituted RAG2^-/-^ mice. (B) Genotyping PCR for floxed and deleted (‘Δ’) alleles of ATMIN and NBS1 performed on DNA from T cells isolated from spleen of ATMIN^ΔL^NBS1^ΔL^ mice.
(TIF)

## References

[1] Dudley DD, Chaudhuri J, Bassing CH, Alt FW (2005) Mechanism and control of V(D)J recombination versus class switch recombination: similarities and differences. Adv Immunol 86: 43–112. 15705419 10.1016/S0065-2776(04)86002-4

[2] Shiloh Y, Ziv Y (2013) The ATM protein kinase: regulating the cellular response to genotoxic stress, and more. Nat Rev Mol Cell Biol 14: 197–210. 10.1038/nrm3546 23847781

[3] Barlow C, Hirotsune S, Paylor R, Liyanage M, Eckhaus M, et al (1996) Atm-deficient mice: a paradigm of ataxia telangiectasia. Cell 86: 159–171. 8689683 10.1016/s0092-8674(00)80086-0

[4] Callen E, Jankovic M, Difilippantonio S, Daniel JA, Chen HT, et al (2007) ATM prevents the persistence and propagation of chromosome breaks in lymphocytes. Cell 130: 63–75. 17599403 10.1016/j.cell.2007.06.016

[5] Camacho E, Hernandez L, Hernandez S, Tort F, Bellosillo B, et al (2002) ATM gene inactivation in mantle cell lymphoma mainly occurs by truncating mutations and missense mutations involving the phosphatidylinositol-3 kinase domain and is associated with increasing numbers of chromosomal imbalances. Blood 99: 238–244. 11756177 10.1182/blood.v99.1.238

[6] Fang NY, Greiner TC, Weisenburger DD, Chan WC, Vose JM, et al (2003) Oligonucleotide microarrays demonstrate the highest frequency of ATM mutations in the mantle cell subtype of lymphoma. Proc Natl Acad Sci U S A 100: 5372–5377. 12697903 10.1073/pnas.0831102100 PMC154352

[7] Haidar MA, Kantarjian H, Manshouri T, Chang CY, O'Brien S, et al (2000) ATM gene deletion in patients with adult acute lymphoblastic leukemia. Cancer 88: 1057–1062. 10699895 10.1002/(sici)1097-0142(20000301)88:5<1057::aid-cncr16>3.0.co;2-6

[8] Schaffner C, Idler I, Stilgenbauer S, Dohner H, Lichter P (2000) Mantle cell lymphoma is characterized by inactivation of the ATM gene. Proc Natl Acad Sci U S A 97: 2773–2778. 10706620 10.1073/pnas.050400997 PMC16005

[9] Stankovic T, Stewart GS, Byrd P, Fegan C, Moss PA, et al (2002) ATM mutations in sporadic lymphoid tumours. Leuk Lymphoma 43: 1563–1571. 12400598 10.1080/1042819021000002884

[10] Stilgenbauer S, Winkler D, Ott G, Schaffner C, Leupolt E, et al (1999) Molecular characterization of 11q deletions points to a pathogenic role of the ATM gene in mantle cell lymphoma. Blood 94: 3262–3264. 10556216

[11] Xu Y, Ashley T, Brainerd EE, Bronson RT, Meyn MS, et al (1996) Targeted disruption of ATM leads to growth retardation, chromosomal fragmentation during meiosis, immune defects, and thymic lymphoma. Genes Dev 10: 2411–2422. 8843194 10.1101/gad.10.19.2411

[12] Zha S, Bassing CH, Sanda T, Brush JW, Patel H, et al (2010) ATM-deficient thymic lymphoma is associated with aberrant tcrd rearrangement and gene amplification. J Exp Med 207: 1369–1380. 10.1084/jem.20100285 20566716 PMC2901073

[13] Ito K, Takubo K, Arai F, Satoh H, Matsuoka S, et al (2007) Regulation of reactive oxygen species by Atm is essential for proper response to DNA double-strand breaks in lymphocytes. J Immunol 178: 103–110. 17182545 10.4049/jimmunol.178.1.103

[14] Barzilai A, Rotman G, Shiloh Y (2002) ATM deficiency and oxidative stress: a new dimension of defective response to DNA damage. DNA Repair (Amst) 1: 3–25. 12509294 10.1016/s1568-7864(01)00007-6

[15] Uziel T, Lerenthal Y, Moyal L, Andegeko Y, Mittelman L, et al (2003) Requirement of the MRN complex for ATM activation by DNA damage. EMBO J 22: 5612–5621. 14532133 10.1093/emboj/cdg541 PMC213795

[16] Carson CT, Schwartz RA, Stracker TH, Lilley CE, Lee DV, et al (2003) The Mre11 complex is required for ATM activation and the G2/M checkpoint. EMBO J 22: 6610–6620. 14657032 10.1093/emboj/cdg630 PMC291825

[17] Stracker TH, Petrini JH (2011) The MRE11 complex: starting from the ends. Nat Rev Mol Cell Biol 12: 90–103. 10.1038/nrm3047 21252998 PMC3905242

[18] Duursma AM, Driscoll R, Elias JE, Cimprich KA (2013) A role for the MRN complex in ATR activation via TOPBP1 recruitment. Mol Cell 50: 116–122. 10.1016/j.molcel.2013.03.006 23582259 PMC3669687

[19] Lee J, Dunphy WG (2013) The Mre11-Rad50-Nbs1 (MRN) complex has a specific role in the activation of Chk1 in response to stalled replication forks. Mol Biol Cell 24: 1343–1353. 10.1091/mbc.E13-01-0025 23468519 PMC3639046

[20] Shiotani B, Nguyen HD, Hakansson P, Marechal A, Tse A, et al (2013) Two distinct modes of ATR activation orchestrated by Rad17 and Nbs1. Cell Rep 3: 1651–1662. 10.1016/j.celrep.2013.04.018 23684611 PMC3680100

[21] Willis N, Rhind N (2010) The fission yeast Rad32(Mre11)-Rad50-Nbs1 complex acts both upstream and downstream of checkpoint signaling in the S-phase DNA damage checkpoint. Genetics 184: 887–897. 10.1534/genetics.109.113019 20065069 PMC2865925

[22] Bruhn C, Zhou ZW, Ai H, Wang ZQ (2014) The essential function of the MRN complex in the resolution of endogenous replication intermediates. Cell Rep 6: 182–195. 10.1016/j.celrep.2013.12.018 24388752

[23] Mazouzi A, Velimezi G, Loizou JI (2014) DNA replication stress: Causes, resolution and disease. Exp Cell Res 329: 85–93. 10.1016/j.yexcr.2014.09.030 25281304

[24] Kracker S, Bergmann Y, Demuth I, Frappart PO, Hildebrand G, et al (2005) Nibrin functions in Ig class-switch recombination. Proc Natl Acad Sci U S A 102: 1584–1589. 15668383 10.1073/pnas.0409191102 PMC547877

[25] Saidi A, Li T, Weih F, Concannon P, Wang ZQ (2010) Dual functions of Nbs1 in the repair of DNA breaks and proliferation ensure proper V(D)J recombination and T-cell development. Mol Cell Biol 30: 5572–5581. 10.1128/MCB.00917-10 20921278 PMC2976431

[26] Reina-San-Martin B, Nussenzweig MC, Nussenzweig A, Difilippantonio S (2005) Genomic instability, endoreduplication, and diminished Ig class-switch recombination in B cells lacking Nbs1. Proc Natl Acad Sci U S A 102: 1590–1595. 15668392 10.1073/pnas.0406289102 PMC547831

[27] Difilippantonio S, Celeste A, Fernandez-Capetillo O, Chen HT, Reina San Martin B, et al (2005) Role of Nbs1 in the activation of the Atm kinase revealed in humanized mouse models. Nat Cell Biol 7: 675–685. 15965469 10.1038/ncb1270

[28] de Miranda NF, Bjorkman A, Pan-Hammarstrom Q (2011) DNA repair: the link between primary immunodeficiency and cancer. Ann N Y Acad Sci 1246: 50–63. 10.1111/j.1749-6632.2011.06322.x 22236430

[29] Kanu N, Behrens A (2007) ATMIN defines an NBS1-independent pathway of ATM signalling. EMBO J 26: 2933–2941. 17525732 10.1038/sj.emboj.7601733 PMC1894771

[30] McNees CJ, Conlan LA, Tenis N, Heierhorst J (2005) ASCIZ regulates lesion-specific Rad51 focus formation and apoptosis after methylating DNA damage. EMBO J 24: 2447–2457. 15933716 10.1038/sj.emboj.7600704 PMC1173145

[31] Schmidt L, Wiedner M, Velimezi G, Prochazkova J, Owusu M, et al (2014) ATMIN is required for the ATM-mediated signaling and recruitment of 53BP1 to DNA damage sites upon replication stress. DNA Repair (Amst). 10.1016/j.dnarep.2014.09.001 PMC4251980 25262557

[32] Kanu N, Penicud K, Hristova M, Wong B, Irvine E, et al (2010) The ATM cofactor ATMIN protects against oxidative stress and accumulation of DNA damage in the aging brain. J Biol Chem 285: 38534–38542. 10.1074/jbc.M110.145896 20889973 PMC2992286

[33] Loizou JI, Sancho R, Kanu N, Bolland DJ, Yang F, et al (2011) ATMIN is required for maintenance of genomic stability and suppression of B cell lymphoma. Cancer Cell 19: 587–600. 10.1016/j.ccr.2011.03.022 21575860 PMC4452547

[34] Jurado S, Gleeson K, O'Donnell K, Izon DJ, Walkley CR, et al (2012) The Zinc-finger protein ASCIZ regulates B cell development via DYNLL1 and Bim. J Exp Med 209: 1629–1639. 10.1084/jem.20120785 22891272 PMC3428950

[35] Zhang T, Penicud K, Bruhn C, Loizou JI, Kanu N, et al (2012) Competition between NBS1 and ATMIN controls ATM signaling pathway choice. Cell Rep 2: 1498–1504. 10.1016/j.celrep.2012.11.002 23219553

[36] Zenewicz LA, Antov A, Flavell RA (2009) CD4 T-cell differentiation and inflammatory bowel disease. Trends Mol Med 15: 199–207. 10.1016/j.molmed.2009.03.002 19362058

[37] Jeggo PA, Lobrich M (2007) DNA double-strand breaks: their cellular and clinical impact? Oncogene 26: 7717–7719. 18066083 10.1038/sj.onc.1210868

[38] Niehues T, Perez-Becker R, Schuetz C (2010) More than just SCID—the phenotypic range of combined immunodeficiencies associated with mutations in the recombinase activating genes (RAG) 1 and 2. Clin Immunol 135: 183–192. 10.1016/j.clim.2010.01.013 20172764

[39] Takahashi N, Matsumoto K, Saito H, Nanki T, Miyasaka N, et al (2009) Impaired CD4 and CD8 effector function and decreased memory T cell populations in ICOS-deficient patients. J Immunol 182: 5515–5527. 10.4049/jimmunol.0803256 19380800

[40] Abolhassani H, Wang N, Aghamohammadi A, Rezaei N, Lee YN, et al (2014) A hypomorphic recombination-activating gene 1 (RAG1) mutation resulting in a phenotype resembling common variable immunodeficiency. J Allergy Clin Immunol 134: 1375–1380. 10.1016/j.jaci.2014.04.042 24996264 PMC4261008

[41] Agarwal S, Smereka P, Harpaz N, Cunningham-Rundles C, Mayer L (2011) Characterization of immunologic defects in patients with common variable immunodeficiency (CVID) with intestinal disease. Inflamm Bowel Dis 17: 251–259. 10.1002/ibd.21376 20629103 PMC3102048

[42] Hartlova A, Erttmann SF, Raffi FA, Schmalz AM, Resch U, et al (2015) DNA damage primes the type I interferon system via the cytosolic DNA sensor STING to promote anti-microbial innate immunity. Immunity 42: 332–343. 10.1016/j.immuni.2015.01.012 25692705

[43] Frappart PO, Tong WM, Demuth I, Radovanovic I, Herceg Z, et al (2005) An essential function for NBS1 in the prevention of ataxia and cerebellar defects. Nat Med 11: 538–544. 15821748 10.1038/nm1228

[44] de Boer J, Williams A, Skavdis G, Harker N, Coles M, et al (2003) Transgenic mice with hematopoietic and lymphoid specific expression of Cre. Eur J Immunol 33: 314–325. 12548562 10.1002/immu.200310005

[45] Nyabi O, Naessens M, Haigh K, Gembarska A, Goossens S, et al (2009) Efficient mouse transgenesis using Gateway-compatible ROSA26 locus targeting vectors and F1 hybrid ES cells. Nucleic Acids Res 37: e55 10.1093/nar/gkp112 19279185 PMC2673446

[46] Vacchio MS, Olaru A, Livak F, Hodes RJ (2007) ATM deficiency impairs thymocyte maturation because of defective resolution of T cell receptor alpha locus coding end breaks. Proc Natl Acad Sci U S A 104: 6323–6328. 17405860 10.1073/pnas.0611222104 PMC1851038

[47] Kolaczkowska E, Kubes P (2013) Neutrophil recruitment and function in health and inflammation. Nat Rev Immunol 13: 159–175. 10.1038/nri3399 23435331

[48] Perse M, Cerar A (2012) Dextran sodium sulphate colitis mouse model: traps and tricks. J Biomed Biotechnol 2012: 718617 10.1155/2012/718617 22665990 PMC3361365

[49] Westbrook AM, Schiestl RH (2010) Atm-deficient mice exhibit increased sensitivity to dextran sulfate sodium-induced colitis characterized by elevated DNA damage and persistent immune activation. Cancer Res 70: 1875–1884. 10.1158/0008-5472.CAN-09-2584 20179206 PMC2831166

[50] Daniel JA, Pellegrini M, Lee BS, Guo Z, Filsuf D, et al (2012) Loss of ATM kinase activity leads to embryonic lethality in mice. J Cell Biol 198: 295–304. 10.1083/jcb.201204035 22869595 PMC3413361

[51] Yamamoto K, Wang Y, Jiang W, Liu X, Dubois RL, et al (2012) Kinase-dead ATM protein causes genomic instability and early embryonic lethality in mice. J Cell Biol 198: 305–313. 10.1083/jcb.201204098 22869596 PMC3413350

[52] Shinkai Y, Rathbun G, Lam KP, Oltz EM, Stewart V, et al (1992) RAG-2-deficient mice lack mature lymphocytes owing to inability to initiate V(D)J rearrangement. Cell 68: 855–867. 1547487 10.1016/0092-8674(92)90029-c

[53] Konca K, Lankoff A, Banasik A, Lisowska H, Kuszewski T, et al (2003) A cross-platform public domain PC image-analysis program for the comet assay. Mutat Res 534: 15–20. 12504751 10.1016/s1383-5718(02)00251-6

[54] Carpenter AE, Jones TR, Lamprecht MR, Clarke C, Kang IH, et al (2006) CellProfiler: image analysis software for identifying and quantifying cell phenotypes. Genome Biol 7: R100 17076895 10.1186/gb-2006-7-10-r100 PMC1794559

