# Supplementary figures and images for: DNA Repair Cofactors ATMIN and NBS1 Are Required to Suppress T Cell Activation
Source: PLoS Genet. 2015 Nov 6;11(11):e1005645. doi: 10.1371/journal.pgen.1005645 (PMC4636180; doi:10.1371/journal.pgen.1005645)

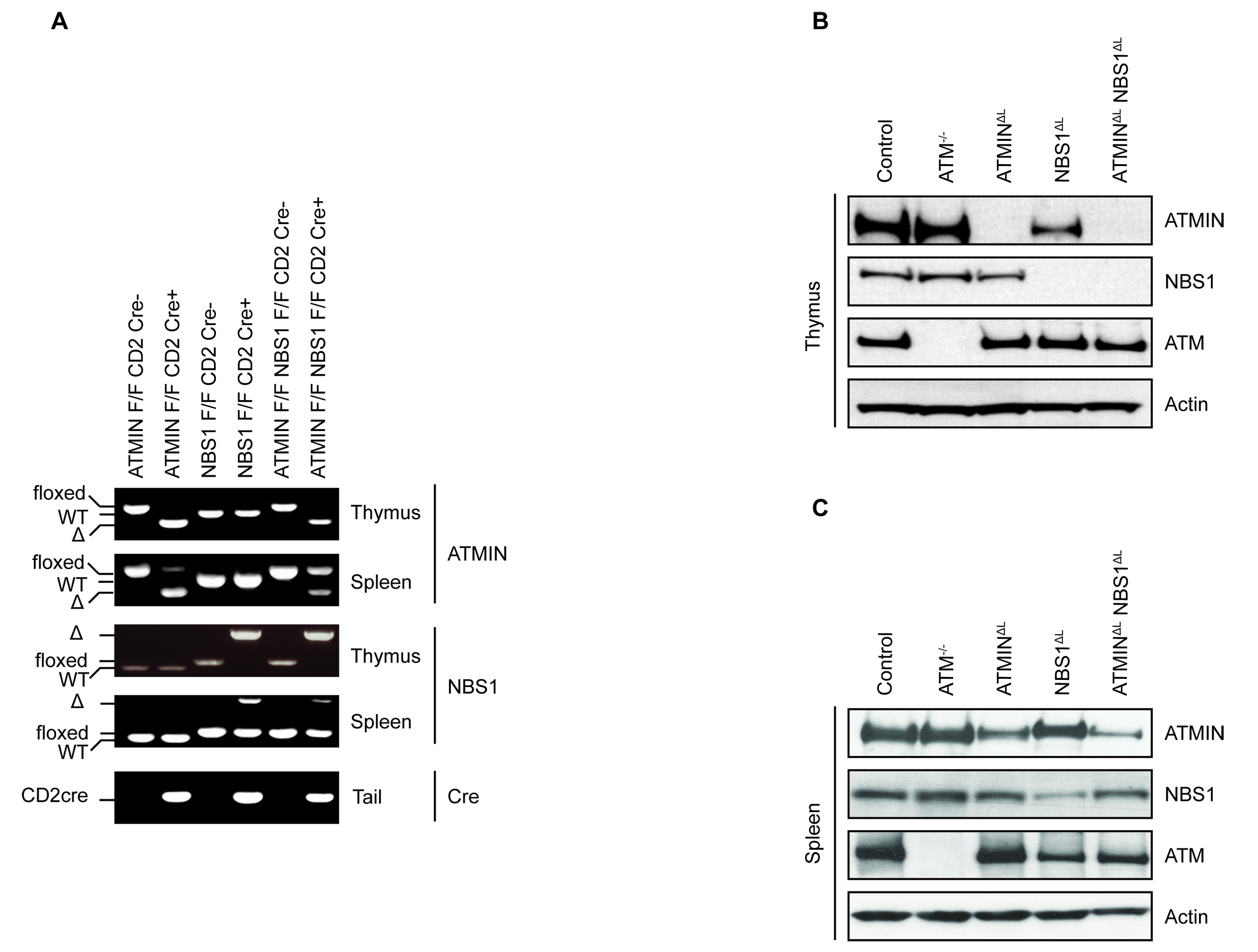

Supplement: S1 Fig — (A) Genotyping PCR for floxed, wild type (‘WT’) and deleted (‘Δ’) alleles of ATMIN and NBS1 performed on DNA from thymus or spleen samples. The PCR for CD2-cre was performed on DNA from tail. Western blot analysis of (B) thymi and (C) spleens from control, ATM-/-, ATMINΔL, NBS1ΔL and ATMINΔLNBS1ΔL mice probed for ATMIN, NBS1, ATM and actin. (TIF) [file pgen.1005645.s001.tif]

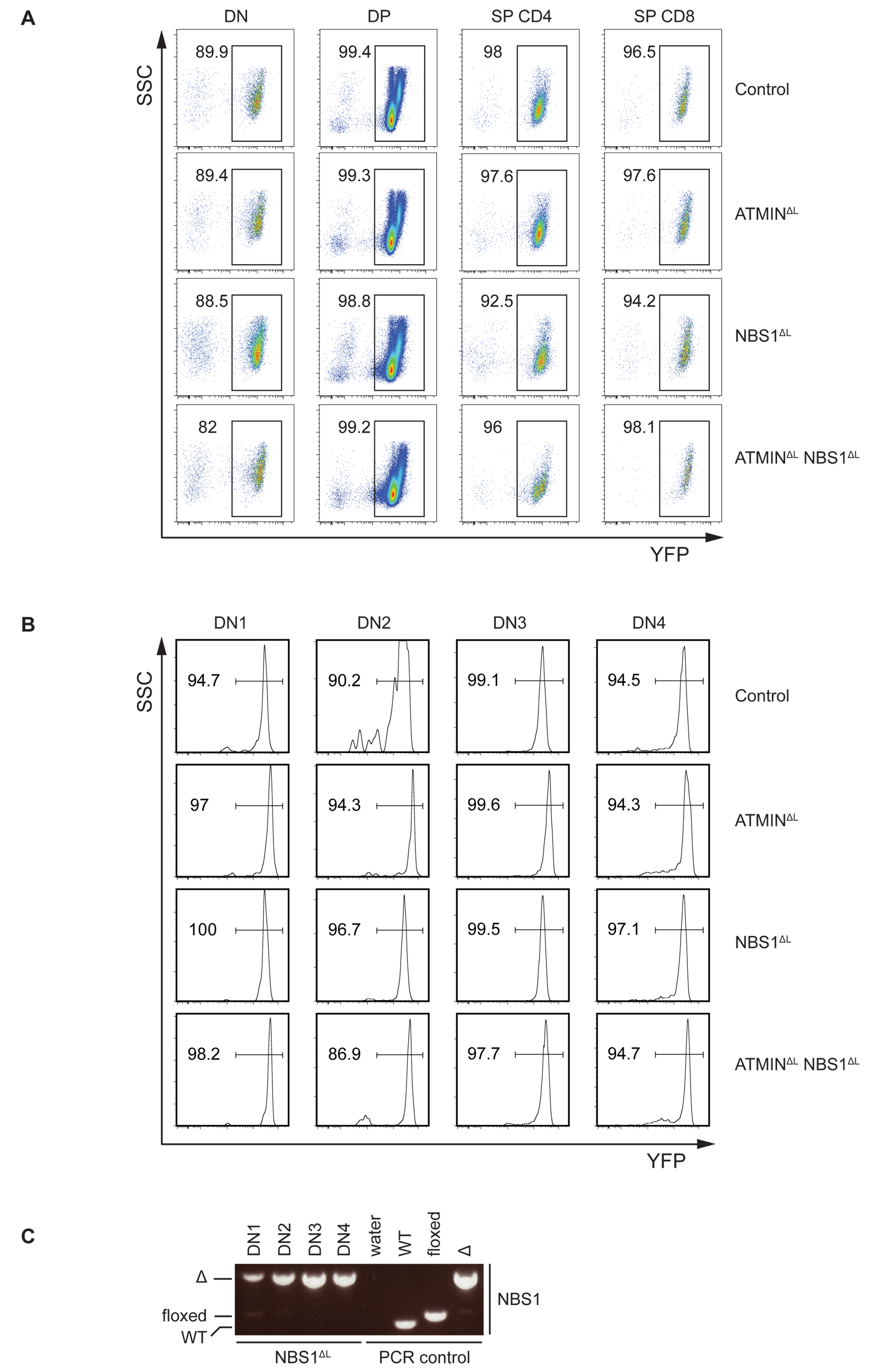

Supplement: S2 Fig — (A) Representative flow cytometry data of YFP expression in DN, DP, SP CD4 and SP CD8 T cells in thymus of control, ATMINΔL, NBS1ΔL and ATMINΔLNBS1ΔL mice. (B) Representative flow cytometry data of YFP expression in DN1-4 T cells in thymus of control, ATMINΔL, NBS1ΔL and ATMINΔLNBS1ΔL mice. (C) Genotyping PCR for floxed, wild type (‘WT’) and deleted (‘Δ’) alleles of NBS1 performed on DNA from DN1-4 T cells from thymus of NBS1ΔL mice. (TIF) [file pgen.1005645.s002.tif]

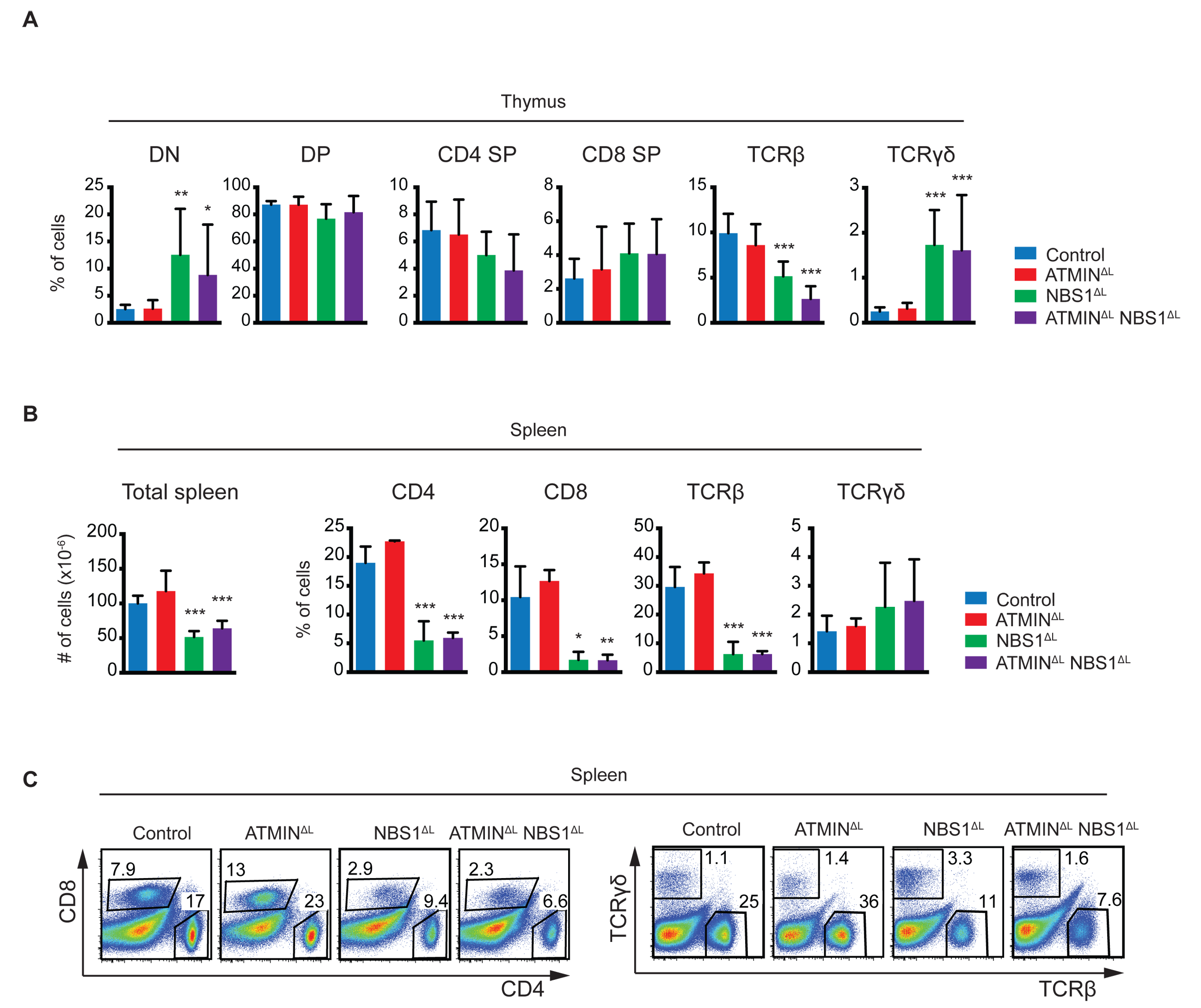

Supplement: S3 Fig — (A) Quantification of T cell subpopulations in the thymus measured by flow cytometry following staining for CD4, CD8, TCRβ and TCRγδ in control, ATMINΔL, NBS1ΔL and ATMINΔLNBS1ΔL mice. N = 4–8 mice per genotype. (B) Quantification of T cell subpopulations in the spleen measured by flow cytometry following staining for CD4, CD8, TCRβ and TCRγδ in control, ATMINΔL, NBS1ΔL and ATMINΔLNBS1ΔL mice. (C) Representative FACS plots of B. N = 3–5 mice per genotype. Error bars represent SEM (*P<0.05, **P<0.01, ***P<0.001). (TIF) [file pgen.1005645.s003.tif]

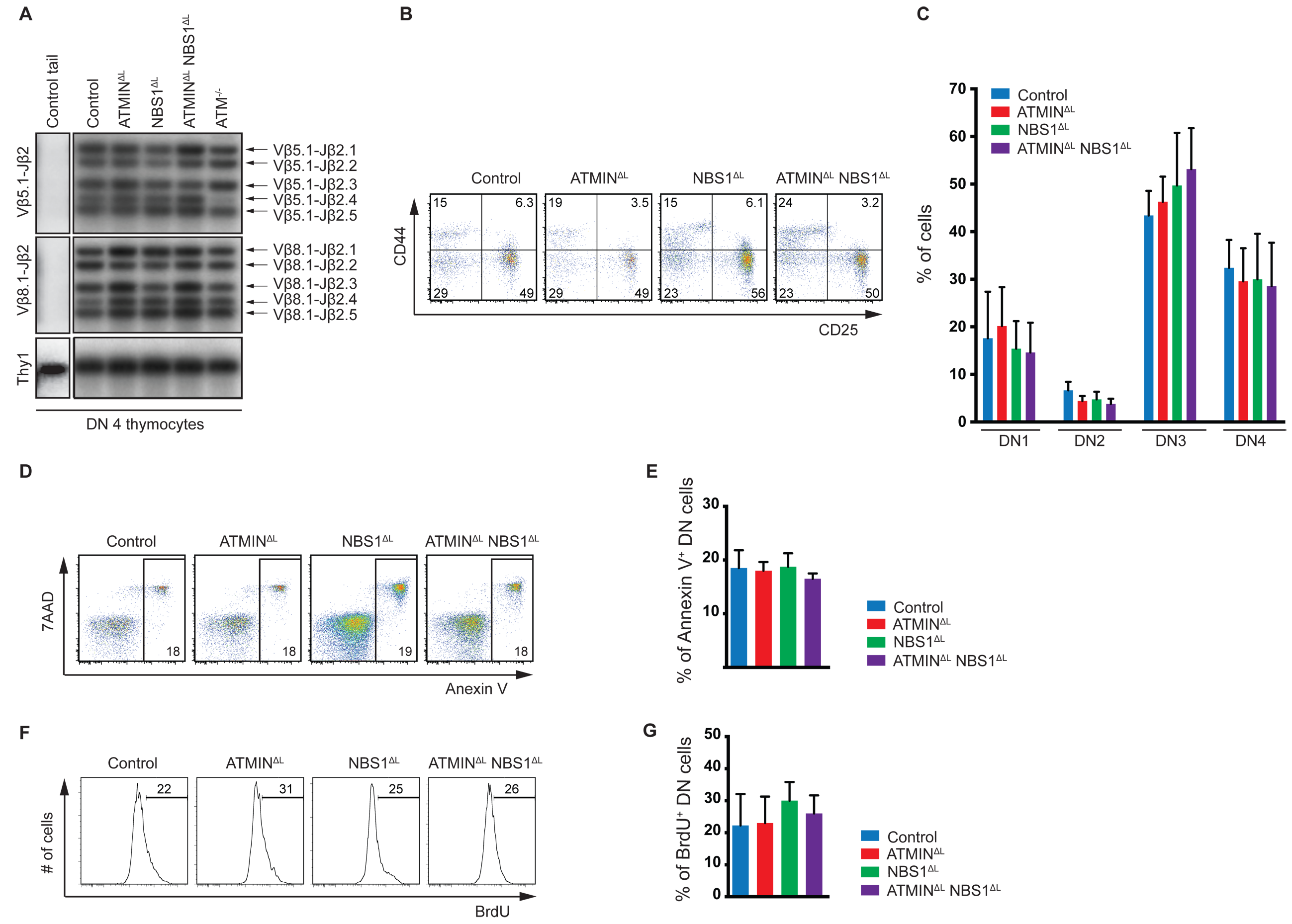

Supplement: S4 Fig — (A) Southern blot analysis of Vβ8.1-Jβ2 and Vβ5.1-Jβ2 recombination regions in FACS-sorted DN4 (CD25-CD44-) thymocytes of control, ATMINΔL, NBS1ΔL and ATMINΔLNBS1ΔL mice. Thy1 is used as a loading control. (B) Representative flow cytometry data of DN1 (CD25-CD44+), DN2 (CD25+CD44+), DN3 (CD25+CD44-) and DN4 (CD25-CD44-) T cell subpopulations in thymi of control, ATMINΔL, NBS1ΔL and ATMINΔLNBS1ΔL mice. N = 5–7 mice per genotype. (C) Quantification of B. (D) Representative flow cytometry data of Annexin V+ apoptotic T cells in DN (CD4-CD8-) population of thymi in mice indicated in A. (E) Quantification of d. N = 4 mice per genotype. (F) Representative flow cytometry results of BrdU+ DN T cells in thymus of mice indicated in A, assessed following 2 days of in vivo BrdU incorporation. (G) Quantification of F. N = 4 mice per genotype. (TIF) [file pgen.1005645.s004.tif]

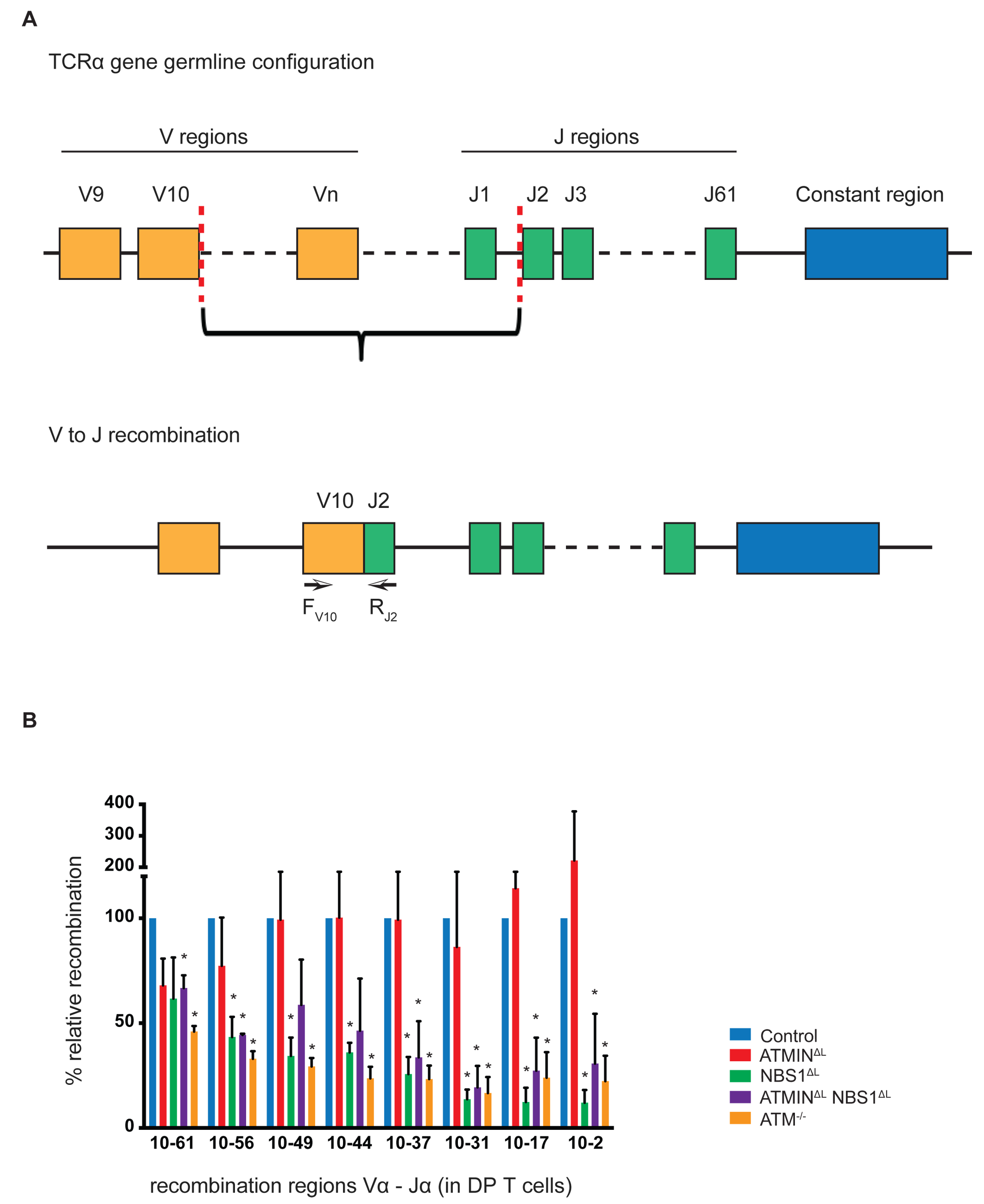

Supplement: S5 Fig — (A) Schematic representation of the V to J recombination events in the TCRα locus. Arrows indicate forward and reverse primers used to amplify the selected V10 to J2 region. (B) Quantitative RT-PCR analysis of eight Vα10-Jα recombination regions in purified DP (CD4+CD8+) thymic T cells from control, ATMINΔL, NBS1ΔL, ATMINΔLNBS1ΔL and ATM-/- mice. Results are normalized to the control DP thymic T cells. N = 3 mice per genotype. Error bars represent SEM (*P<0.05). (TIF) [file pgen.1005645.s005.tif]

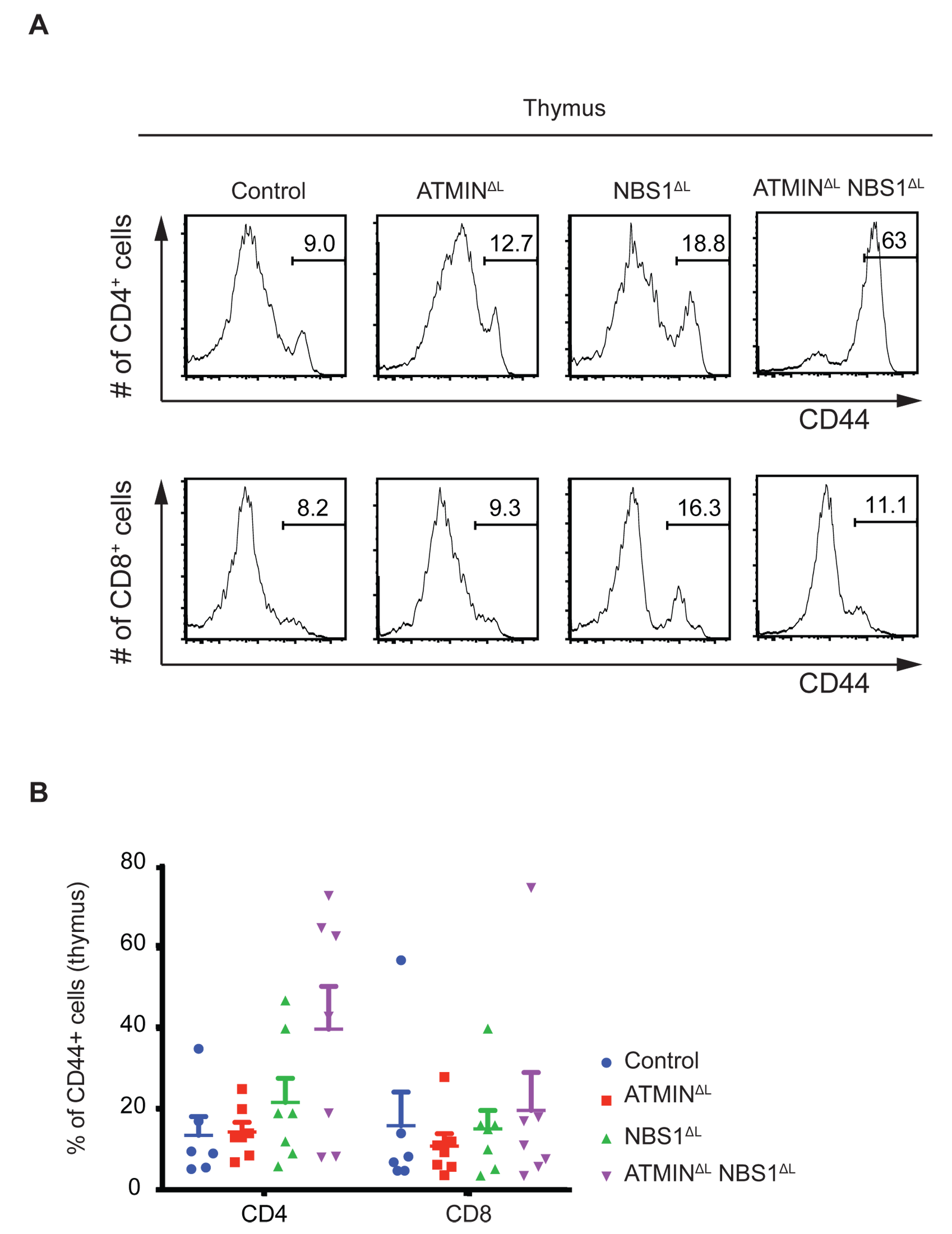

Supplement: S6 Fig — (A) Representative flow cytometry data of CD44 expression on CD4+ and CD8+ thymocytes in control, ATMINΔL, NBS1ΔL and ATMINΔLNBS1ΔL mice. (B) Quantification of A. (TIF) [file pgen.1005645.s006.tif]

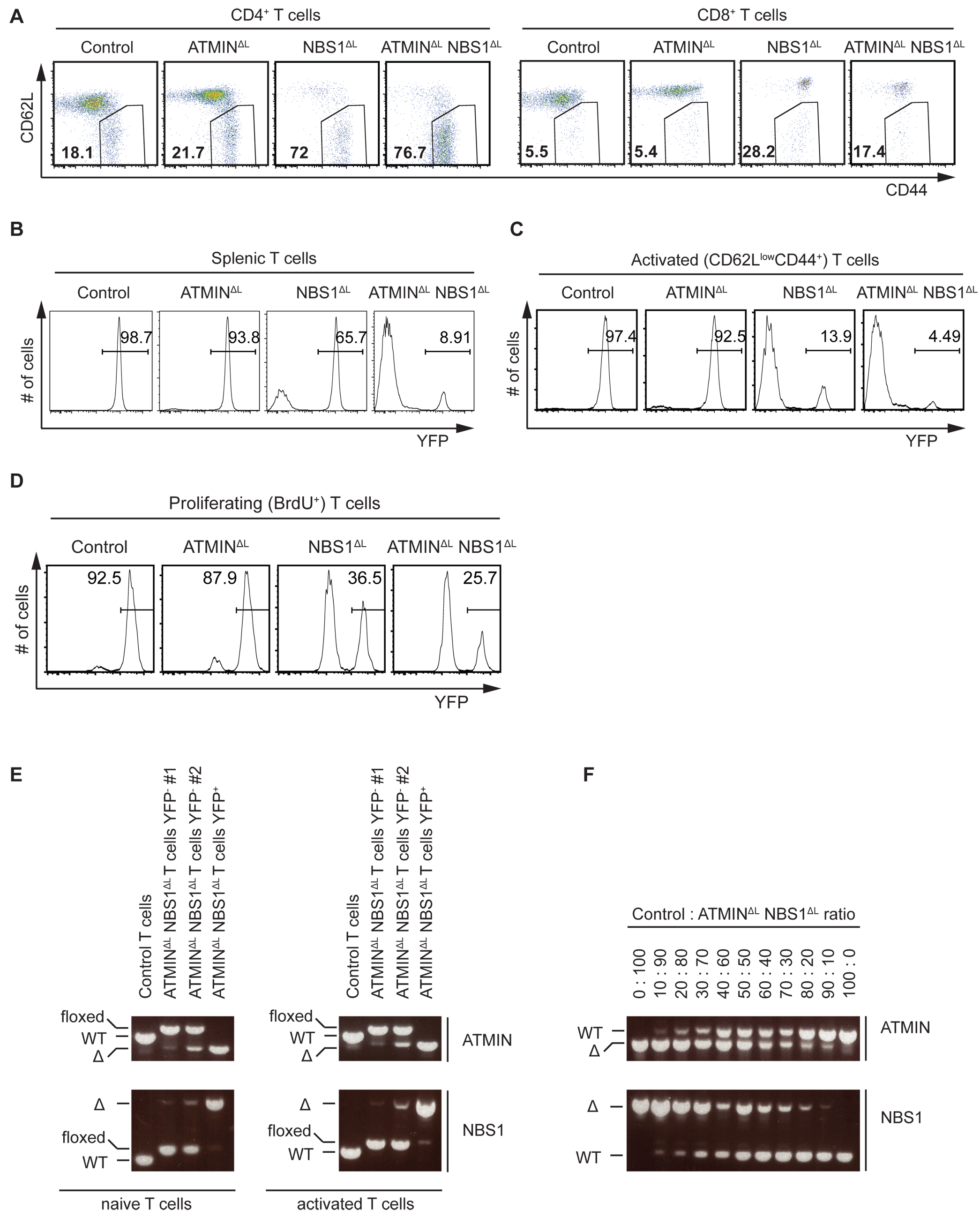

Supplement: S7 Fig — (A) Representative flow cytometry data of activated (CD62LlowCD44+) CD4+ and CD8+ T cells in the spleen of control, ATMINΔL, NBS1ΔL and ATMINΔLNBS1ΔL mice. (B) Representative flow cytometry data of YFP+ T cells (gated on the TCRβ+ population) in the spleen of control, ATMINΔL, NBS1ΔL and ATMINΔLNBS1ΔL mice. (C) Representative flow cytometry data of YFP+ activated (CD44+CD62Llow) T cells in the spleen of control, ATMINΔL, NBS1ΔL and ATMINΔLNBS1ΔL mice. (D) Representative flow cytometry data of YFP+ proliferating (BrdU+) T cells in the spleen of control, ATMINΔL, NBS1ΔL and ATMINΔLNBS1ΔL mice. (E) Genotyping PCR of control, two biological replicates of YFP- ATMINΔLNBS1ΔL and YFP+ ATMINΔLNBS1ΔL FACS sorted (for TCRβ+ populations) splenic T cells, without TCR stimulation (‘naïve T cells’) or with TCR stimulation (‘activated T cells’). (F) Genotyping PCR of dilution series of control versus ATMINΔLNBS1ΔL T cells from the thymus. (TIF) [file pgen.1005645.s007.tif]

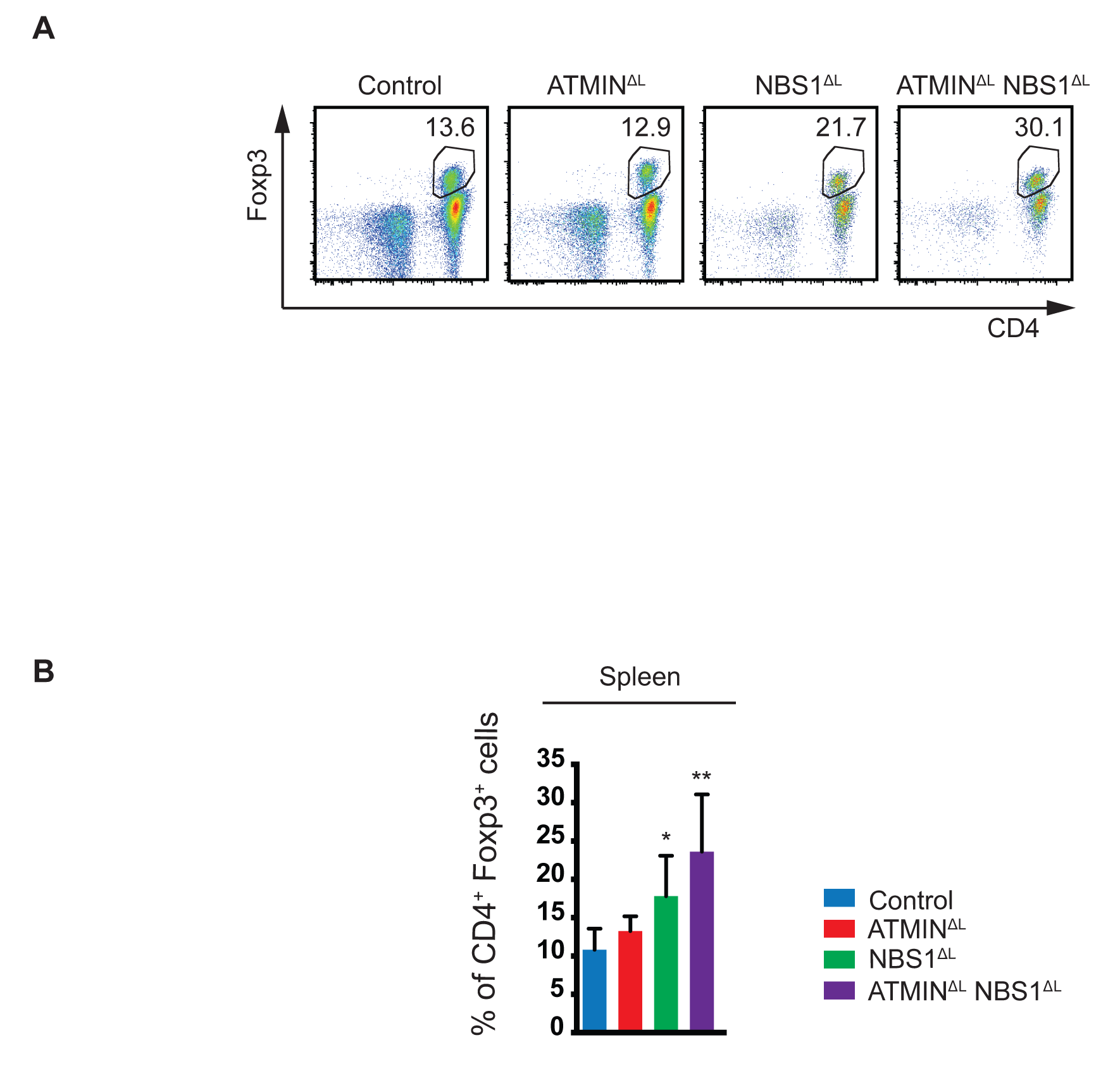

Supplement: S8 Fig — (A) Representative flow cytometry data of CD4+Foxp3+ regulatory T (gated on the TCRβ+ population) cells in the spleen of control, ATMINΔL, NBS1ΔL, ATMINΔLNBS1ΔL and ATM-/- mice. N = 5–7 mice per genotype. (B) Quantification of A. Error bars represent SEM (*P<0.05, **P<0.01). (TIF) [file pgen.1005645.s008.tif]

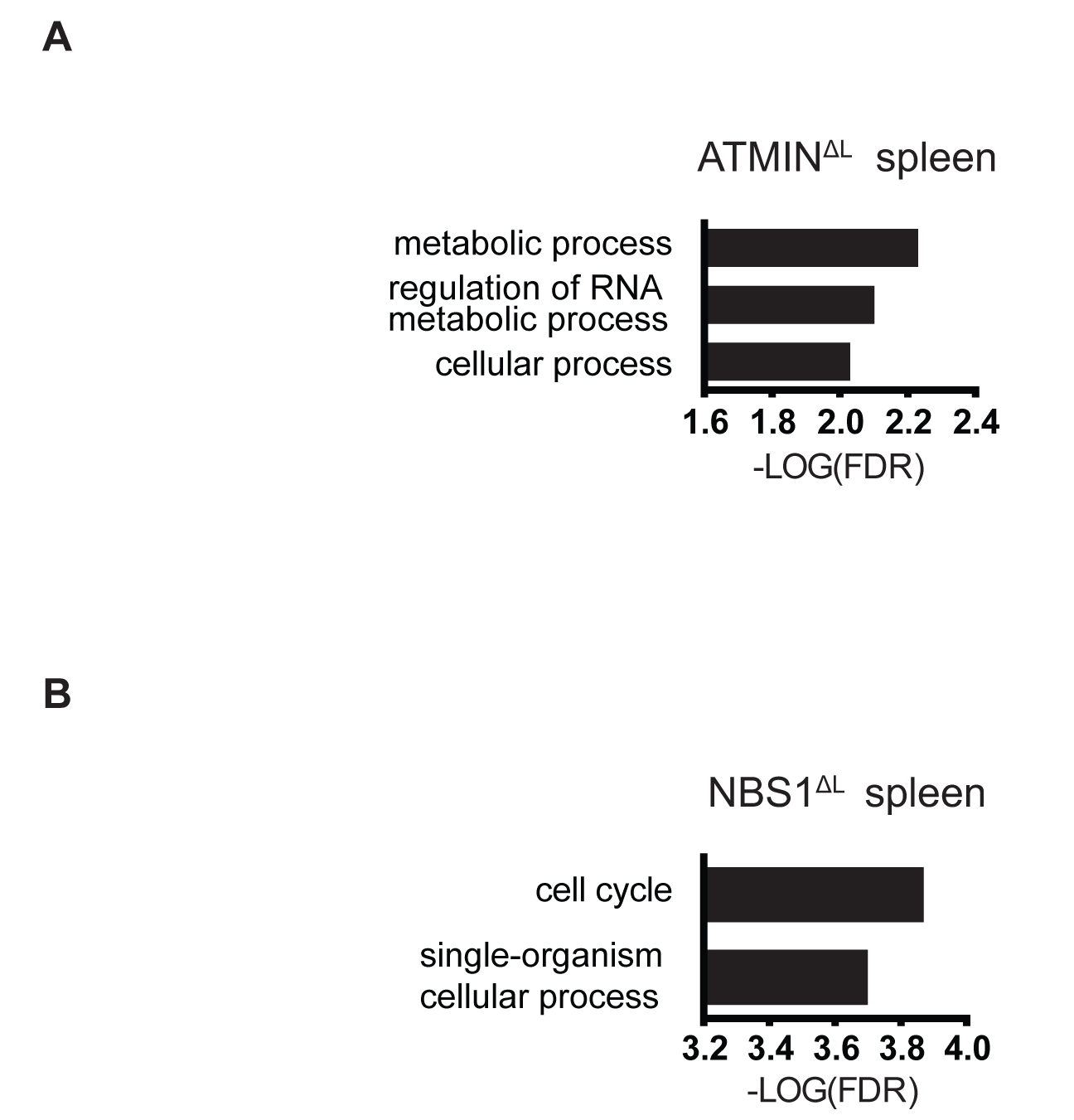

Supplement: S9 Fig — (A) Gene ontology analysis of processes enriched in spleen of ATMINΔL mice identified by mRNA-sequencing. (B) Gene ontology analysis of processes enriched in spleen of NBS1ΔL mice identified by mRNA-sequencing. (TIF) [file pgen.1005645.s009.tif]

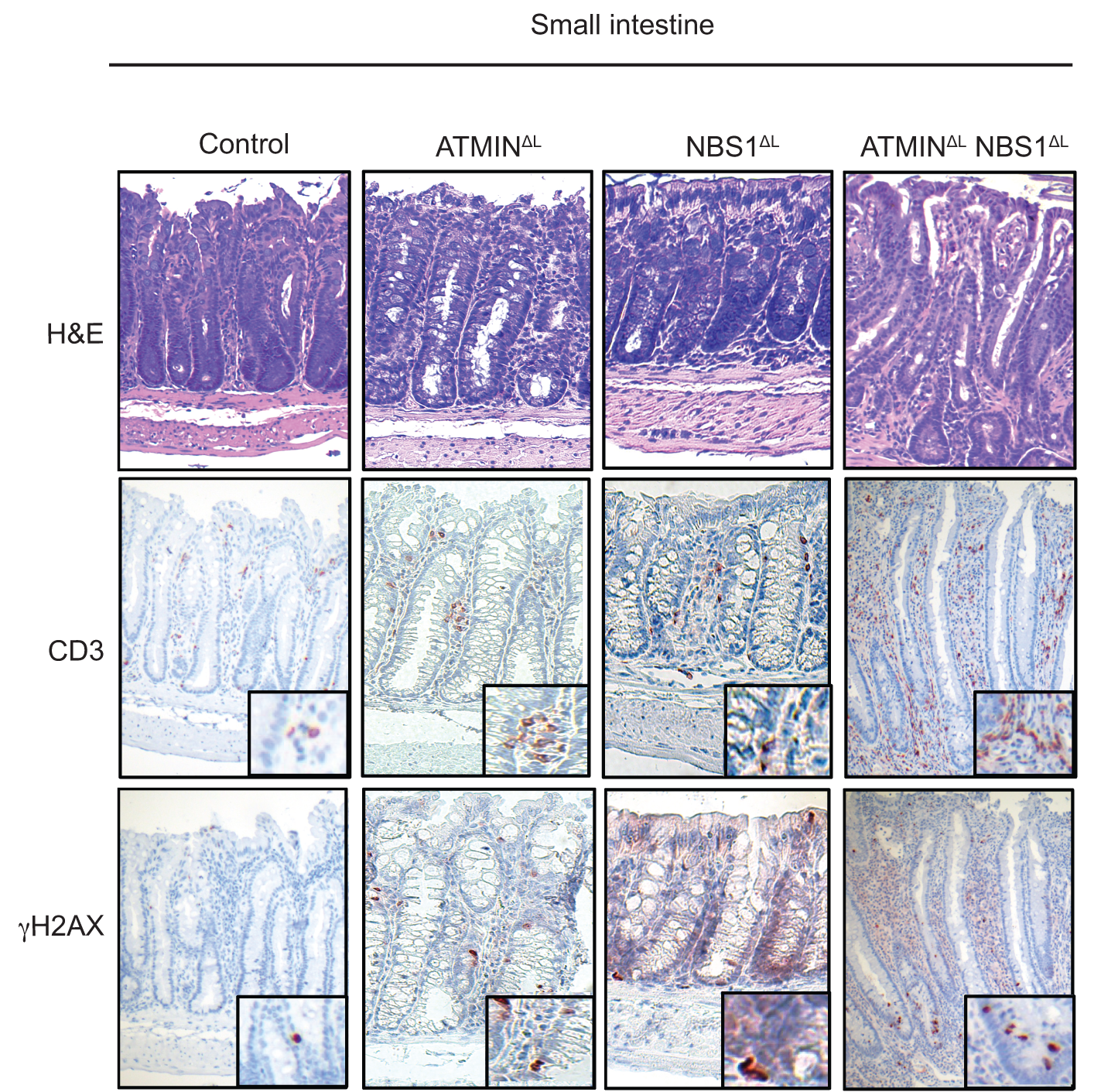

Supplement: S10 Fig — Intestines of control, ATMINΔL, NBS1ΔL and ATMINΔLNBS1ΔL mice were stained by H&E and for γH2AX as well as CD3. (TIF) [file pgen.1005645.s010.tif]

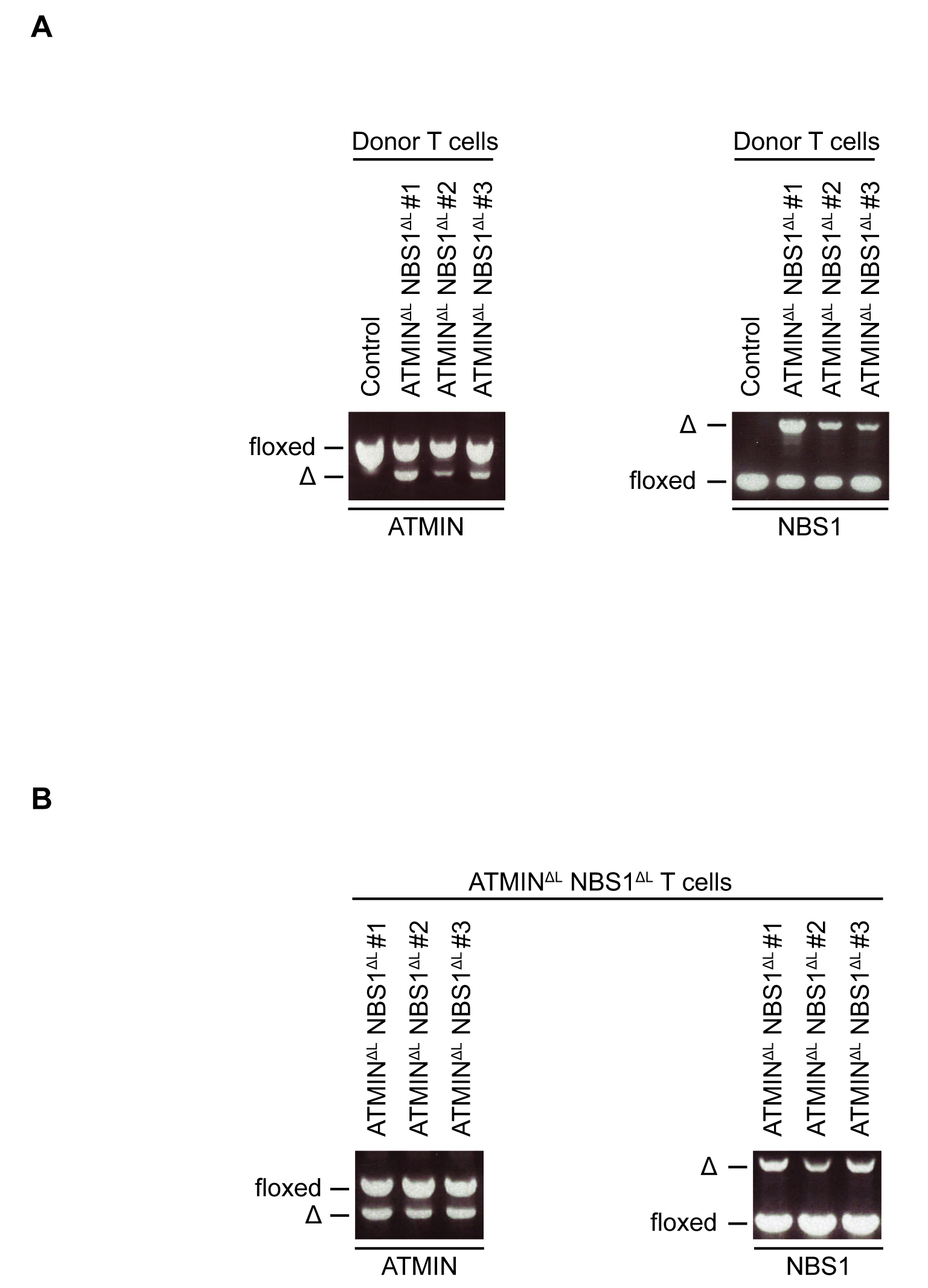

Supplement: S11 Fig — (A) Genotyping PCR for floxed and deleted (‘Δ’) alleles of ATMIN and NBS1 was performed on DNA from FACS sorted (TRCβ+ populations) T cells isolated from reconstituted RAG2-/- mice. (B) Genotyping PCR for floxed and deleted (‘Δ’) alleles of ATMIN and NBS1 performed on DNA from T cells isolated from spleen of ATMINΔLNBS1ΔL mice. (TIF) [file pgen.1005645.s011.tif]
